# Thromboinflammatory and Pharmacological Effects of Low-Molecular-Weight Heparins in Acute Venous Thromboembolism: An Integrated Clinical and In Silico Analysis

**DOI:** 10.3390/medsci14020260

**Published:** 2026-05-19

**Authors:** Lutfi Cagatay Onar, Ersin Guner, Irem Ozten Dalkiran, Ibrahim Yilmaz

**Affiliations:** 1 Department of Cardiovascular Surgery, Dr. Ismail Fehmi Cumalioglu City Hospital, Republic of Turkey Ministry of Health, Tekirdag 59020, Turkey; 2 Department of Pharmacy, Konya Numune Hospital, Republic of Turkey Ministry of Health, Konya 42060, Turkey; 3 Department of Midwifery, University of Health Sciences, Istanbul 34668, Turkey; 4 Unit of Pharmacovigilance, Dr. Ismail Fehmi Cumalioglu City Hospital, Republic of Turkey Ministry of Health, Tekirdag 59020, Turkey

**Keywords:** disease enrichment, low-molecular-weight heparin, molecular docking, network pharmacology, protein–protein interaction, thromboinflammation, tinzaparin, venous thromboembolism

## Abstract

**Background:** Venous thromboembolism (VTE) is a thromboinflammatory disorder involving coordinated activation of coagulation, endothelial dysfunction, and inflammatory signaling. Low-molecular-weight heparins (LMWHs) may exert pharmacological effects beyond anticoagulation. This study compared enoxaparin, bemiparin, and tinzaparin and explored potential multi-target mechanisms using molecular docking, network pharmacology, and enrichment analyses. **Methods:** In this retrospective cohort study, patients with acute VTE treated with therapeutic-dose LMWHs were analyzed. Stabilized IPTW based on multinomial propensity scores was used to reduce baseline imbalance between treatment groups. Clinical recovery was assessed using the Clinical Severity Score (CSS). Thromboinflammatory biomarkers (MPV, hs-CRP, NLR, fibrinogen) were evaluated during follow-up. Molecular docking, STRING/Cytoscape-based protein–protein interaction, and enrichment analyses were performed. **Results:** Median time to symptom resolution was 31 days with enoxaparin, 28 days with bemiparin, and 24 days with tinzaparin (log-rank *p* < 0.001). Recovery was faster with bemiparin (HR 1.28, 95% CI 1.05–1.56) and tinzaparin (HR 1.72, 95% CI 1.41–2.10). Tinzaparin showed greater reductions in hs-CRP, MPV, NLR, and fibrinogen (all *p* < 0.05) and less analgesic use beyond 10 days (19.7% vs. 27.0% and 33.2%; *p* < 0.001). Docking analyses identified plausible conformations (root-mean-square deviation, RMSD ≤ 2 Å). Given the structural flexibility and heterogeneous chain length of LMWHs, rigid docking algorithms may not fully capture biologically relevant conformations. Therefore, docking results should be interpreted as qualitative interaction mapping rather than quantitative binding affinity estimation. Network analysis highlighted F3, TNF, IL6, and VWF, while enrichment analyses suggested involvement of cytokine signaling, leukocyte migration, and thromboinflammatory pathways. **Conclusions:** LMWH therapy was associated with improved thromboinflammatory markers and clinical recovery, with tinzaparin showing comparatively more favorable thromboinflammatory biomarker trajectories and recovery dynamics within the limitations of this observational analysis. Integrated clinical and in silico findings provide hypothesis-generating insights into potential multi-target pharmacological effects beyond anticoagulation; however, these observations should be interpreted cautiously and require experimental validation.

## 1. Introduction

Venous thromboembolism (VTE) is increasingly recognized as a thromboinflammatory condition in which coagulation activation, platelet reactivity, endothelial dysfunction, and inflammatory signaling interact through closely interconnected biological pathways. The formation of venous thrombus is accompanied by leukocyte recruitment, cytokine release, and endothelial activation, creating a local and systemic inflammatory milieu that contributes to thrombus propagation and delayed clinical recovery. This thromboinflammatory paradigm has shifted therapeutic considerations beyond anticoagulation alone toward interventions capable of modulating both hemostatic and inflammatory pathways.

Low-molecular-weight heparins (LMWHs) remain cornerstone agents in the treatment of acute VTE. Their principal pharmacodynamic effect involves potentiation of antithrombin-mediated inhibition of factor Xa and, to a lesser extent, thrombin. However, increasing evidence indicates that LMWHs exert pleiotropic biological effects beyond anticoagulation. These include modulation of cytokine signaling, leukocyte adhesion, platelet activation, and endothelial function. Network pharmacology and in silico analyses have demonstrated that heparin interacts with multiple inflammatory and coagulation-related targets, supporting a multi-target pharmacological profile rather than a single-pathway anticoagulant mechanism [1]. These findings provide mechanistic support for the anti-inflammatory effects observed during LMWH therapy.

Platelet-mediated mechanisms appear central to the thromboinflammatory activity modulated by heparins. Platelet factor 4 (PF4), released from activated platelets, binds strongly to heparin oligosaccharides, forming multimolecular complexes that influence platelet-driven inflammatory signaling. Structural studies have shown that long heparin chains interact with PF4 to form ultralarge complexes, providing a plausible structural basis for platelet–heparin immune interactions [2]. These interactions may affect leukocyte recruitment, endothelial activation, and cytokine release, thereby linking platelet activation to inflammatory signaling.

Heparin derivatives also interact with growth factor signaling systems involved in endothelial repair and vascular remodeling. Experimental and in silico studies indicate that heparin-induced angiogenesis may be mediated through basic fibroblast growth factor-2, suggesting a role for heparin in vascular healing and thrombus resolution [3]. Endothelial restoration is particularly relevant in VTE, where endothelial injury contributes to both thrombus formation and persistent inflammatory signaling. In addition, glycosaminoglycan–protein binding studies emphasize that heparin interactions with extracellular matrix-associated proteins influence inflammatory and vascular responses [4].

Deep vein thrombosis (DVT), the most common clinical manifestation of venous thromboembolism, represents a state of intense thromboinflammatory activation in which fibrin deposition, platelet aggregation, endothelial perturbation, and leukocyte recruitment collectively contribute to both macrovascular obstruction and microvascular perfusion impairment. Although systemic thrombolytic therapy is not indicated in most patients with uncomplicated DVT, therapeutic administration of LMWHs may facilitate endogenous fibrinolytic activity. This biological framework supports the concept that anticoagulant treatment in acute DVT may function not only to prevent thrombus propagation but also to facilitate physiologic thrombolytic cascades and thromboinflammatory resolution. Accordingly, modulation of thromboinflammatory signaling may influence both thrombus stabilization and the tempo of clinical recovery in acute VTE.

Molecular docking studies further suggest potential multi-target pharmacological interactions of LMWHs. Computational analyses have suggested that enoxaparin may form stable interactions with diverse protein complexes in silico [5]. Similarly, investigations of heparin–protein interactions show that structural binding to plasma proteins may influence anticoagulant efficacy and pharmacodynamic variability [6]. In addition to docking, network-based analyses and enrichment approaches have further highlighted multi-target effects of LMWHs. These findings should be interpreted as exploratory computational associations rather than direct mechanistic evidence.

Emerging mechanistic evidence further suggests that heparin-binding regulatory proteins may contribute to the regulation of coagulation–inflammation crosstalk. For example, latexin, a protein involved in coagulation and inflammation, has been shown to interact with heparin and influence thrombotic pathways [7]. In addition, structural studies examining interactions between heparin derivatives and cytokine-related growth factors indicate that molecular composition affects protein binding stability and biological activity [8]. Such variability may contribute to pharmacodynamic differences among LMWH formulations.

Additional investigations of glycosaminoglycan–protein interactions highlight the importance of sulfation patterns and chain length in modulating interactions with inflammatory mediators [4]. These structural determinants may contribute to pharmacodynamic variability and heterogeneous clinical responses among LMWH formulations. Despite these mechanistic insights, head-to-head clinical data on the biological response profiles of individual LMWH agents remain limited.

Enoxaparin, bemiparin, and tinzaparin differ in molecular weight distribution, anti-Xa to anti-IIa activity ratios, and affinity for endothelial and platelet-associated proteins. These pharmacological differences may translate into measurable variability in biological response among formulations. Integration of clinical observations with in silico modeling may therefore help contextualize potential pharmacodynamic variability among LMWH formulations. Molecular docking and network-based analyses may further help identify ligand–protein interactions and coordinated coagulation–inflammation networks potentially associated with these agents [9].

Accordingly, the present study compared the clinical and biomarker effects of enoxaparin, bemiparin, and tinzaparin in patients with acute venous thromboembolism and explored potential molecular mechanisms underlying observed clinical differences using integrated molecular docking and network- and enrichment-based in silico analyses. By combining clinical biomarker assessment with computational evaluation of protein interactions, the study provides an exploratory translational context regarding potential pharmacodynamic differences in LMWH therapy.

Longitudinal biomarker trajectories, time-to-recovery analyses, and mediation-based approaches were integrated to explore potential associations between biomarker dynamics and clinical recovery across the three LMWH agents.

## 2. Materials and Methods

### 2.1. Study Design and Ethical Approval

This retrospective observational cohort study included consecutive patients with objectively confirmed acute venous thromboembolism treated with therapeutic-dose LMWHs between January 2014 and December 2020. Treatment allocation was determined by the treating physician according to clinical judgment and local drug availability, reflecting real-world prescribing patterns. The analytical strategy was therefore designed to account for baseline imbalances between treatment groups.

The study was conducted in accordance with the Declaration of Helsinki and approved by the institutional ethics committee (approval number AN-260403-03, dated 4 March 2026). Due to the retrospective design, the requirement for written informed consent was waived.

Eligible patients were adults with objectively confirmed acute venous thromboembolism who received therapeutic-dose enoxaparin, bemiparin, or tinzaparin as initial anticoagulant therapy. A total of 921 consecutive patients were included in the final cohort (enoxaparin *n* = 307, bemiparin *n* = 304, tinzaparin *n* = 310).

### 2.2. Data Acquisition

Clinical, laboratory, and imaging data were retrieved retrospectively from the hospital electronic medical record system, laboratory information database, and radiology archive. Baseline demographic characteristics and predefined clinical risk factors were recorded.

Information regarding thromboembolic presentation, treatment allocation, and timing of therapy initiation was obtained from archived clinical documentation. Laboratory measurements at predefined follow-up time points and serial clinical severity assessments were extracted from structured outpatient visit records. Only patients with complete baseline and follow-up data were included in the final analytical cohort. Patients with active infection, chronic inflammatory or autoimmune disorders, and hematological conditions potentially affecting biomarker interpretation were excluded.

### 2.3. Diagnostic Confirmation of Venous Thromboembolism

Venous thromboembolism was confirmed using objective imaging modalities according to established diagnostic standards. Deep vein thrombosis was diagnosed by compression duplex ultrasonography, demonstrating non-compressibility of the affected venous segment, visualization of intraluminal thrombus, or absence of normal venous flow patterns. Pulmonary embolism was confirmed by computed tomography pulmonary angiography, demonstrating intraluminal filling defects within the pulmonary arterial circulation.

Patients with contraindications to LMWH therapy, treatment discontinuation at baseline, or incomplete laboratory and follow-up clinical assessments were excluded from the final analysis.

### 2.4. Treatment Protocol

All patients received therapeutic-dose LMWH therapy as first-line anticoagulant therapy initiated after objective confirmation of venous thromboembolism. Treatment allocation reflected routine clinical decision-making based on physician preference, patient characteristics, and institutional drug availability, and was not influenced by the study protocol. Patients received weight-adjusted therapeutic dosing of enoxaparin, bemiparin, or tinzaparin according to institutional practice and contemporary clinical recommendations.

### 2.5. Clinical Follow-Up and Outcome Assessment

Clinical evaluations were performed at predefined follow-up time points on Days 0, 7, 20, 30, and 45 after treatment initiation. Assessments were conducted during outpatient visits or structured telephone follow-up, documented within the hospital electronic medical record system. Day 0 corresponded to the time of initial presentation and represented the point of maximum symptom severity. Follow-up assessments included evaluation of symptom burden, functional limitation, analgesic requirement, and longitudinal changes in thromboinflammatory biomarkers.

#### 2.5.1. Clinical Severity Score

Clinical symptom burden was quantified using a structured composite Clinical Severity Score (CSS) ranging from 0 to 10, with higher values indicating greater symptom severity. The score incorporated pain intensity, limb swelling, erythema, warmth, and functional limitation. Measurements were obtained at each predefined follow-up visit to characterize symptom improvement and recovery dynamics.

#### 2.5.2. Definition of Recovery Trajectories

Recovery trajectories were evaluated using time-dependent changes in CSS values across follow-up assessments. Functional recovery was defined as the first time point at which a CSS of 2 or lower was achieved and maintained without subsequent deterioration. Complete recovery was defined as a CSS of 0. Time to functional recovery was selected as the primary clinical outcome. Time to complete symptom resolution and duration of analgesic requirement beyond 10 days were analyzed as secondary outcomes. Associations between early biomarker changes and clinical improvement were explored.

### 2.6. Laboratory Measurements

Laboratory measurements were obtained at baseline and repeated on Days 7, 20, and 45 after treatment initiation. Evaluated parameters included mean platelet volume (MPV), high-sensitivity C-reactive protein (hs-CRP), erythrocyte sedimentation rate (ESR), neutrophil-to-lymphocyte ratio (NLR), platelet-to-lymphocyte ratio (PLR), and fibrinogen concentration. Serial biomarker measurements were incorporated into longitudinal analyses to assess thromboinflammatory activity and its relationship with clinical recovery.

### 2.7. Propensity Score Weighting

Because treatment allocation was not randomized, stabilized inverse probability of treatment weighting (IPTW) based on multinomial propensity scores was applied to reduce baseline imbalances between treatment groups. Propensity scores were estimated using multinomial logistic regression incorporating prespecified demographic, clinical, thromboembolic, and thromboinflammatory covariates.

Covariate balance after weighting was assessed using absolute standardized mean differences (SMD), with values below 0.10 considered indicative of acceptable balance. Positivity and overlap assumptions were additionally evaluated by visual inspection of propensity score distributions across treatment groups. Detailed propensity score diagnostics are provided in the Supplementary Materials.

### 2.8. Longitudinal Biomarker Modeling

Longitudinal changes in thromboinflammatory biomarkers were analyzed using linear mixed-effects regression models including treatment group, follow-up time, and treatment-by-time interaction terms. Random effects were specified at the patient level to account for repeated measurements. Inverse probability of treatment weighting and prespecified baseline covariates were incorporated into the models.

### 2.9. Mediation Analysis

To evaluate whether early changes in thromboinflammatory biomarkers mediated treatment-associated differences in clinical recovery, causal mediation analyses were performed within a regression-based counterfactual framework. Early biomarker changes between baseline and Day 7 were evaluated as candidate mediators for time to functional recovery. Models incorporated inverse probability of treatment weighting and prespecified baseline covariates.

### 2.10. Early Biomarker Response Analysis

Early biomarker response was evaluated using relative changes in thromboinflammatory markers between baseline and Day 7. Associations between early biomarker reduction and time to functional recovery were analyzed using inverse probability–weighted Cox regression models adjusted for prespecified baseline covariates. Interaction and Kaplan–Meier analyses were performed to explore differences in recovery dynamics.

### 2.11. Subgroup and Sensitivity Analyses

Prespecified subgroup analyses were conducted according to thrombus localization, presence of pulmonary embolism, baseline inflammatory burden, and inherited thrombophilia. Additional sensitivity analyses were performed across demographic and clinical variables to assess the robustness of treatment-associated recovery estimates.

Detailed methodological information, including eligibility criteria, variable definitions, treatment specifications, scoring criteria, laboratory procedures, and extended statistical modeling approaches (propensity score weighting, longitudinal modeling, mediation analysis, early biomarker response analysis, and subgroup analyses), is provided in the Supplementary Materials.

### 2.12. Statistical Analysis

Continuous variables were assessed for normality using the Kolmogorov–Smirnov test. Normally distributed variables were expressed as mean ± standard deviation, whereas non-normally distributed variables were reported as median with interquartile range. Categorical variables were summarized as counts and percentages.

Baseline comparisons among treatment groups were performed using one-way analysis of variance or the Kruskal–Wallis test, as appropriate. Post hoc pairwise comparisons were conducted with Bonferroni correction.

Time-to-event outcomes were evaluated using Kaplan–Meier survival analysis and compared with the log-rank test. Hazard ratios with 95% confidence intervals were estimated using Cox proportional hazards regression models incorporating inverse probability weighting.

Associations between early biomarker changes and clinical recovery were evaluated using weighted Cox regression models. Longitudinal biomarker trajectories were analyzed using mixed-effects regression models. A two-tailed *p* value < 0.05 was considered statistically significant.

Statistical analyses were performed using SPSS Statistics version 20.0 (IBM Corp., Armonk, New York, NY, USA) and R version 4.3.1 (R Foundation for Statistical Computing, Vienna, Austria).

### 2.13. In Silico Analyses

#### 2.13.1. Molecular Docking Workflow

To complement the clinical findings and explore potential molecular mechanisms underlying thromboinflammatory modulation, molecular docking analyses were performed using a standardized computational workflow. Ligand structures of enoxaparin, bemiparin, and tinzaparin were generated and energy-minimized prior to docking, and receptor preparation included removal of crystallographic water molecules, addition of polar hydrogens, and assignment of atomic charges. Docking simulations were conducted using AutoDock 4.2.6 with the Lamarckian genetic algorithm, and ten independent docking runs were performed for each receptor–ligand pair. Binding energies, hydrogen bond interactions, and root-mean-square deviation (RMSD) values were evaluated, and conformations with RMSD ≤ 2 Å were considered stable docking solutions. Ligand preparation, receptor preprocessing, software configuration, and docking parameters are described in the Supplementary Materials.

Docking analyses were conducted against key thromboinflammatory targets, including thrombin exosite I (PDB ID: 5E8E) [10], thrombin exosite II (PDB ID: 3B9F) [11], P-selectin (PDB ID: 1G1S) [12], IL-6 receptor (PDB ID: 1P9M) [13], PF4 (PDB ID: 4R9W) [14], tissue factor (PDB ID: 1DAN) [15], ICAM-1 (PDB ID: 1IC1) [16], and VCAM-1 (PDB ID: 1IJ9) [17]. For each protein, receptor preparation, grid definition, and docking parameters were standardized, and ligand–protein complexes were evaluated according to binding energy, hydrogen-bond interactions, and RMSD values. Receptor preparation steps, grid coordinates, and docking parameters for each target are provided in the Supplementary Materials.

#### 2.13.2. Target Selection Strategy

Target selection for downstream network analysis followed a docking-driven pharmacological rationale. The present study did not employ RNA sequencing or transcriptomic datasets; instead, proteins with established roles in coagulation, platelet activation, endothelial adhesion, and inflammatory signaling were selected a priori for docking. After evaluation of docking interactions, the target list was subsequently expanded to include closely related proteins identified within the interaction network to capture the broader thromboinflammatory context. The final protein set comprised F10, F2, F3, ICAM1, IL6, IL6R, PF4, SELP, SERPINC1, TLR4, TNF, VCAM1, and VWF. This approach preserved mechanistic consistency with the docking analysis while enabling systems-level interpretation of pharmacological effects. Because the network was constructed using docking-derived targets, the analysis should be considered hypothesis-driven rather than discovery-based, and enrichment findings primarily provide contextual biological support.

#### 2.13.3. STRING Protein Interaction Analysis

A protein–protein interaction (PPI) analysis was performed using the STRING database (version 12.0; https://string-db.org; accessed on 13 May 2026) with *Homo sapiens* selected as the organism and a minimum interaction score of 0.700 (high confidence) [18]. The 13-gene set derived from docking-based target selection was uploaded, and interactions were evaluated using experimental evidence, curated databases, and functional association data. Enrichment analysis was performed to identify significantly overrepresented Gene Ontology biological processes and Kyoto Encyclopedia of Genes and Genomes pathways related to coagulation and inflammation.

#### 2.13.4. Functional Enrichment Analysis

Functional enrichment analyses were performed within the STRING environment [18], including Gene Ontology (GO) biological process (BP), molecular function (MF), and cellular component (CC) categories, as well as Kyoto Encyclopedia of Genes and Genomes (KEGG) pathway analysis. Enrichment results were filtered to retain biologically relevant processes associated with coagulation, inflammation, platelet activation, endothelial function, and vascular signaling. Disease-specific enrichment terms that emerged due to shared inflammatory mediators, such as infection-related pathways, were not incorporated into the final biological interpretation to maintain mechanistic relevance to venous thromboembolism.

#### 2.13.5. Cytoscape Network Construction

The STRING-derived PPI network was exported as tab-separated values (TSV) files and imported into Cytoscape software (version 3.10.4; https://cytoscape.org; accessed on 13 May 2026) [19] for downstream visualization and network analysis. Network topology was evaluated using the cytoHubba plugin [20]. Cytoscape was used to reconstruct the interaction network, enabling identification of functional clusters linking coagulation factors, cytokine signaling mediators, adhesion molecules, and platelet-related proteins. This network-level visualization enabled exploratory integration of docking-derived targets within a thromboinflammatory signaling framework.

#### 2.13.6. Hub Gene Prioritization

Identification of hub genes was performed using the cytoHubba plugin in Cytoscape. Four topological algorithms-maximal clique centrality (MCC), Degree, Betweenness, and Closeness centrality-were applied to evaluate node importance within the PPI network. Genes consistently ranked highly across MCC, and the centrality metrics were considered key hub genes. This multi-algorithm approach was adopted to minimize bias associated with single-metric centrality analysis and to improve robustness of hub gene selection. The analysis was performed using interactions derived from STRING with a minimum required interaction score of 0.700 (high confidence) [20].

#### 2.13.7. Disease Association Enrichment 

Disease enrichment analysis was performed using the Enrichr platform [21] (https://maayanlab.cloud/Enrichr/; accessed on 13 May 2026) to identify disease associations of docking-derived thromboinflammatory targets. The final 13-gene set (F10, F2, F3, ICAM1, IL6, IL6R, PF4, SELP, SERPINC1, TLR4, TNF, VCAM1, and VWF) was uploaded to the Enrichr web server. Disease enrichment results were retrieved from the DisGeNET [22] and Jensen DISEASES curated databases [23]. Terms were ranked according to adjusted *p*-values, and the top enriched disease categories associated with thromboinflammatory and cardiovascular conditions were selected for interpretation. Bar graph visualizations were generated directly from the Enrichr output. Enrichment results were used to provide contextual biological relevance of the identified targets in thromboinflammatory disease mechanisms. These disease associations reflect shared inflammatory mediators and should not be interpreted as independent mechanistic validation.

#### 2.13.8. Functional and Phenotype Enrichment Analysis

To further validate functional relevance and clinical associations, additional enrichment analyses were performed using GO Biological Process and Human Phenotype Ontology libraries available in the Enrichr platform. These analyses were conducted to identify inflammatory biological processes and clinically relevant thromboembolic phenotypes associated with the docking-derived targets. Terms were ranked according to adjusted *p*-values, and the top enriched terms were visualized using bar plots.

## 3. Results

### 3.1. Study Population and Baseline Characteristics

A total of 1164 consecutive patients with objectively confirmed venous thromboembolism were screened for eligibility. After application of predefined inclusion and exclusion criteria, 921 patients were included in the final analytic cohort, comprising 307 patients treated with enoxaparin, 304 treated with bemiparin, and 310 treated with tinzaparin. Baseline demographic and clinical characteristics before weighting are summarized in Table 1. Covariate balance diagnostics and numerical SMD values before and after weighting are presented in Supplementary Figure S2 and Supplementary Table S1, respectively.

**Figure 1:**
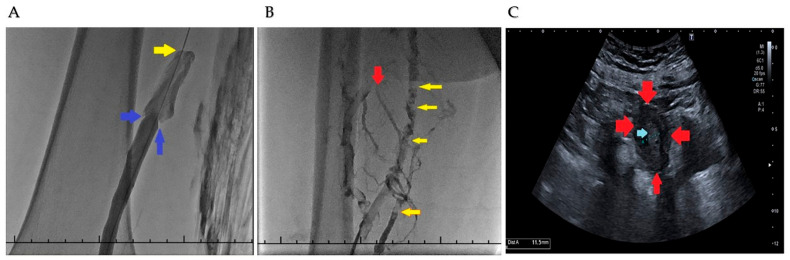
Representative fluoroscopic and ultrasonographic findings in medically less responsive lower-extremity deep vein thrombosis. (**A**) Fluoroscopic image obtained during venographic assessment. Blue arrows indicate intraluminal valve-like structures or flow-related filling irregularities located approximately 4 cm proximal to the junction of the left popliteal vein and common femoral vein. Turbulent flow secondary to thrombus burden is observed in the venous segment proximal to these structures, with complete cessation of contrast passage at the level of the occlusive thrombus. The yellow arrow denotes the anatomical site of total luminal occlusion. (**B**) Fluoroscopic image demonstrating extensive thrombus burden within the common femoral vein with collateral venous filling. Yellow arrows indicate collateral venous channels, whereas the red arrow marks the region of thrombotic obstruction. This representative panel illustrates advanced venous outflow impairment in a patient with insufficient symptomatic regression under LMWH therapy. (**C**) Lower-extremity venous Doppler ultrasonographic image of the proximal one-third of the right common femoral vein in a patient with complicated and recurrent venous thromboembolism. Red arrows delineate the outer vascular wall, whereas the blue arrow identifies centrally located occlusive thromboembolic material measuring at least 11.5 mm in diameter. The vessel demonstrates marked dilatation with layered thrombus of heterogeneous echogenicity, compatible with thromboembolic material of different apparent chronicity along the venous wall. Additional fluoroscopic examples are provided in Supplementary Figure S1.

Clinical presentation reflected the expected symptomatic spectrum of acute venous thromboembolism. Unilateral limb swelling was observed in 702 patients (76.2%), followed by localized pain in 681 patients (73.9%). Erythema and increased local warmth were documented in 412 patients (44.7%), and functional limitation was present in 366 patients (39.7%). Low-grade fever accompanied the clinical presentation in 148 patients (16.1%), whereas dyspnea or pleuritic chest pain suggestive of pulmonary embolism was reported in 257 patients (27.9%). Among patients with pulmonary embolism, 71 individuals demonstrated concomitant proximal lower extremity thrombosis. Representative fluoroscopic and ultrasonographic imaging findings illustrating thrombus burden, collateral development, and disease progression in medically less responsive lower extremity deep vein thrombosis are shown in Figure 1.

Predisposing clinical conditions associated with the index thromboembolic event included postoperative thrombosis, immobilization-related thrombosis, obesity-associated thrombosis, hormone therapy-related thrombosis, and postpartum thrombosis, with similar distributions across treatment groups (Table 2). A history of contralateral deep vein thrombosis occurring more than five years before the index event and inherited thrombophilia, including Factor V Leiden or homozygous MTHFR mutation, were present in a minority of patients and were retained for prespecified subgroup analyses. Because patients with active malignancy were excluded according to study design, the variable previous malignancy refers to malignancy in remission at the time of index venous thromboembolism. Predisposing clinical conditions, including immobilization, recent surgery, obesity-related thrombosis, malignancy, and hormone therapy, are summarized in Table 2.

### 3.2. Covariate Balance After Propensity Score Weighting

To reduce baseline treatment selection bias and improve comparability across treatment groups, inverse probability of treatment weighting was applied using a multinomial propensity score framework incorporating prespecified baseline covariates. Variables included age, sex, body mass index, diabetes mellitus, hypertension, smoking status, proximal deep vein thrombosis involvement, presence of pulmonary embolism, baseline hs-CRP concentration, previous contralateral deep vein thrombosis (>5 years), previous pulmonary embolism history, inherited thrombophilia (Factor V Leiden mutation or homozygous MTHFR mutation), previous malignancy in remission, and postpartum venous thrombosis. After weighting, covariate balance improved substantially. Standardized mean differences for all baseline variables were below 0.10, indicating adequate balance and supporting the validity of subsequent weighted outcome analyses. Visual inspection of standardized mean difference distributions before and after weighting confirmed effective reduction in baseline imbalance across demographic characteristics, thrombus localization variables, inflammatory markers, and thrombotic risk modifiers included in the propensity score model.

### 3.3. CSS Framework and Symptom Dynamics

Clinical symptom burden during follow-up was quantified using the CSS, a composite measure ranging from 0 to 10 that integrates pain intensity, limb swelling, local inflammatory signs including erythema and warmth, functional limitation, and systemic symptoms. Assessments were performed at predefined time points on Days 0, 7, 20, 30, and 45. Baseline CSS values reflected moderate to marked thromboinflammatory symptom burden across treatment groups and did not differ significantly between groups at presentation. Progressive reductions in CSS values were observed during follow-up in all treatment groups, consistent with the gradual resolution of thromboinflammatory activity during anticoagulant therapy.

**Figure 2:**
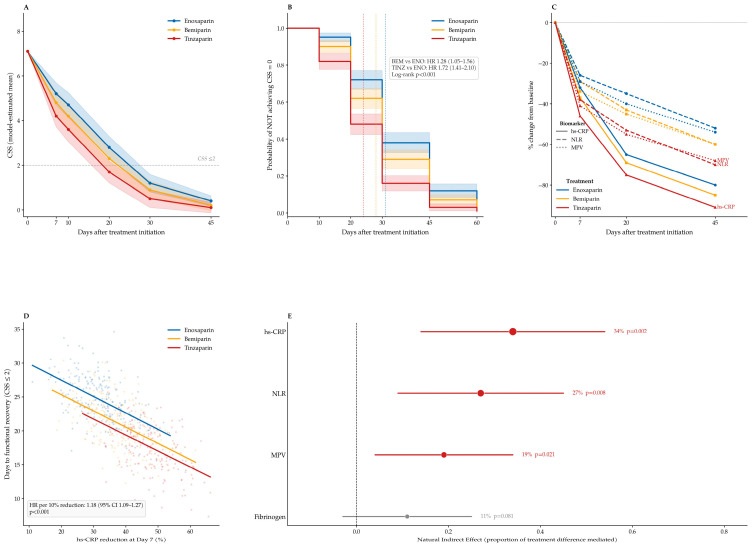
Integrated clinical recovery trajectories and thromboinflammatory response patterns during LMWH therapy. (**A**) Model-estimated longitudinal trajectories of CSS across treatment groups. (**B**) Kaplan–Meier curves for time to functional recovery. (**C**) Percent change from baseline in thromboinflammatory biomarkers during follow-up. (**D**) Association between early reduction in hs-CRP and time to functional recovery. (**E**) Mediation analysis showing the proportion of treatment effect explained by early biomarker reductions. Error bars, shaded regions, or horizontal lines represent 95% confidence intervals where applicable.

Clinically meaningful improvement, defined as a reduction of at least 50 percent in CSS, occurred earlier in patients treated with tinzaparin compared with bemiparin and enoxaparin. Similarly, the achievement of functional recovery, defined as a CSS of 2 or lower, occurred earlier in the tinzaparin group, supporting treatment-associated differences in symptom resolution trajectories. Temporal evolution of CSS values across treatment groups is illustrated in Figure 2A, demonstrating progressive symptom improvement throughout the observation period, with earlier separation of recovery curves observed in the tinzaparin group. Definitions and component structure of the CSS are summarized in Table 3.

### 3.4. Clinical Recovery Trajectories According to LMWH Treatment

Clinical recovery trajectories differed significantly across treatment groups during follow-up. Kaplan–Meier curves for time to functional recovery (CSS ≤ 2) are shown in Figure 2B. Median time to complete symptom resolution, defined as achievement of a CSS of 0, was 31 days in the enoxaparin group, 28 days in the bemiparin group, and 24 days in the tinzaparin group (log-rank *p* < 0.001; Table 4).

Inverse probability-weighted Cox proportional hazards regression analysis confirmed faster recovery in patients treated with tinzaparin compared with enoxaparin (hazard ratio 1.72, 95% confidence interval 1.41 to 2.10, *p* < 0.001) and in patients treated with bemiparin compared with enoxaparin (hazard ratio 1.28, 95% confidence interval 1.05 to 1.56, *p* = 0.014). Recovery trajectories also remained significantly shorter with tinzaparin compared with bemiparin (hazard ratio 1.34, 95% confidence interval 1.11 to 1.62, *p* = 0.002).

Kaplan–Meier analysis demonstrated early separation of recovery curves within the first week after treatment initiation, with persistent divergence throughout the observation period.

Analysis of predefined CSS thresholds demonstrated consistent treatment-associated differences across multiple recovery endpoints (Table 5).

Time to mild symptom status, defined as a CSS of 5 or lower, occurred at 12.1 ± 3.6 days in the enoxaparin group, 10.3 ± 3.2 days in the bemiparin group, and 8.4 ± 2.9 days in the tinzaparin group (*p* < 0.001). Functional recovery, defined as a CSS of 2 or lower, occurred at 24.3 ± 5.1 days, 21.2 ± 4.7 days, and 17.6 ± 4.1 days, respectively (*p* < 0.001). Complete symptom resolution, defined as a CSS of 0, was also achieved earlier in patients treated with tinzaparin than in those receiving enoxaparin (*p* < 0.001) or bemiparin (*p* = 0.006).

### 3.5. Longitudinal Changes in Thromboinflammatory Biomarkers

Longitudinal trajectories of thromboinflammatory biomarkers demonstrated progressive reductions across all treatment groups during follow-up, with greater declines observed in patients treated with tinzaparin compared with bemiparin and enoxaparin.

Mixed-effects regression models confirmed significant treatment-by-time interactions for hs-CRP, MPV, NLR, PLR, ESR, and fibrinogen (all *p* interaction ≤ 0.006). These results were consistent with differential temporal patterns of thromboinflammatory resolution across treatment groups.

Detailed regression coefficients and slope differences derived from mixed-effects models are presented in Table 6, and longitudinal biomarker trajectories are illustrated in Figure 2C. Associations between early hs-CRP reduction and recovery time, together with exploratory mediation analyses, are shown in Figure 2D,E.

### 3.6. Early Biomarker Response and Prediction of Functional Recovery

Early reductions in thromboinflammatory biomarkers between baseline and Day 7 were significantly associated with subsequent clinical recovery trajectories. Patients demonstrating greater early reductions in hs-CRP, NLR, MPV, and fibrinogen concentrations achieved functional recovery earlier than patients with smaller biomarker changes.

Inverse probability-weighted Cox proportional hazards models incorporating early biomarker response demonstrated that larger relative reductions in hs-CRP were independently associated with shorter time to functional recovery (hazard ratio per 10 percent reduction 1.18, 95 percent confidence interval 1.09 to 1.27, *p* < 0.001). Similar associations were observed for NLR (hazard ratio 1.14, 95 percent confidence interval 1.06 to 1.22, *p* = 0.002) and MPV (hazard ratio 1.11, 95 percent confidence interval 1.03 to 1.19, *p* = 0.006).

Kaplan–Meier analyses stratified according to tertiles of early hs-CRP reduction demonstrated progressive shortening of recovery time across response strata (log-rank *p* < 0.001). These associations remained consistent across prespecified clinical subgroups, including thrombus localization, presence of pulmonary embolism, inherited thrombophilia status, postpartum venous thrombosis, and previous contralateral deep vein thrombosis history.

When evaluated jointly in multivariable inverse probability-weighted models, early reductions in hs-CRP remained the strongest independent biomarker predictor of functional recovery. Importantly, early divergence of biomarker trajectories observed within the first seven days of treatment was concordant with longitudinal mixed-effects model estimates.

These findings suggest that early attenuation of thromboinflammatory activity may represent a clinically relevant intermediate pattern associated with downstream recovery dynamics. The magnitude and temporal pattern of early inflammatory decline suggested that high-sensitivity CRP may represent a plausible candidate biomarker associated with accelerated functional recovery. Early percent reductions in thromboinflammatory biomarkers between baseline and early follow-up and their associations with time to functional recovery are summarized in Table 7.

### 3.7. Biomarker-Mediated Associations with Functional Recovery

Causal mediation analyses were performed to explore associations between early reductions in thromboinflammatory biomarkers and treatment-associated differences in clinical recovery. Early changes between baseline and Day 7 in hs-CRP, NLR, MPV, and fibrinogen were evaluated as candidate mediators. Given the observational design, mediation findings should be interpreted as exploratory associations rather than causal effects.

Early reduction in hs-CRP was associated with the strongest mediation effect. Approximately 34% of the treatment-associated difference in recovery observed between tinzaparin and enoxaparin was statistically associated with early reduction in hs-CRP (*p* < 0.001).

Smaller but significant mediation effects were observed for NLR (27%, *p* = 0.002) and MPV (19%, *p* = 0.006), whereas fibrinogen showed no significant indirect effect. Combined models incorporating multiple biomarkers suggested that early changes in hs-CRP and NLR were associated with treatment-associated differences in functional recovery.

### 3.8. Subgroup Analyses

Prespecified subgroup analyses were performed according to thrombus localization, presence of pulmonary embolism, baseline inflammatory burden, inherited thrombophilia, postpartum venous thrombosis, and previous contralateral deep vein thrombosis.

Treatment-associated differences in time to functional recovery remained directionally consistent across all subgroups. Faster recovery with tinzaparin compared with bemiparin and enoxaparin was observed irrespective of thrombus localization, presence of pulmonary embolism, or baseline inflammatory burden. Similar patterns were noted in patients with and without inherited thrombophilia, postpartum venous thrombosis, and prior contralateral deep vein thrombosis.

No statistically significant treatment-by-subgroup interactions were detected, indicating consistent treatment effects across clinically relevant thromboinflammatory phenotypes.

### 3.9. Sensitivity Analyses

Sensitivity analyses were performed according to sex, smoking status, and previous malignancy in remission. Treatment-associated differences in time to functional recovery remained consistent across all strata. Faster recovery with tinzaparin compared with bemiparin and enoxaparin was observed in both sexes, in smokers and non-smokers, and in patients with or without previous malignancy. No significant treatment-by-subgroup interactions were detected. Alternative model specifications and additional covariate adjustments yielded similar estimates, supporting robustness of the primary findings.

### 3.10. Safety Outcomes and Rescue Interventions

LMWH therapy was well tolerated across treatment groups, with no clinically meaningful differences in major bleeding events (Table 5). Analgesic requirement beyond Day 10 was less frequent in patients treated with tinzaparin compared with bemiparin and enoxaparin (19.7%, 27.0%, and 33.2%, respectively; *p* < 0.001). Recurrent symptoms during follow-up were infrequent and numerically lower with tinzaparin, without statistically significant differences. Major bleeding events were rare and comparable across treatment groups, and no fatal bleeding events were observed. Details regarding rescue interventions, including catheter-directed thrombolysis and inferior vena cava filter placement, are summarized in Supplementary Section S3.

### 3.11. Clinical Presentation and Visual Spectrum of Disease Severity

**Figure 3:**
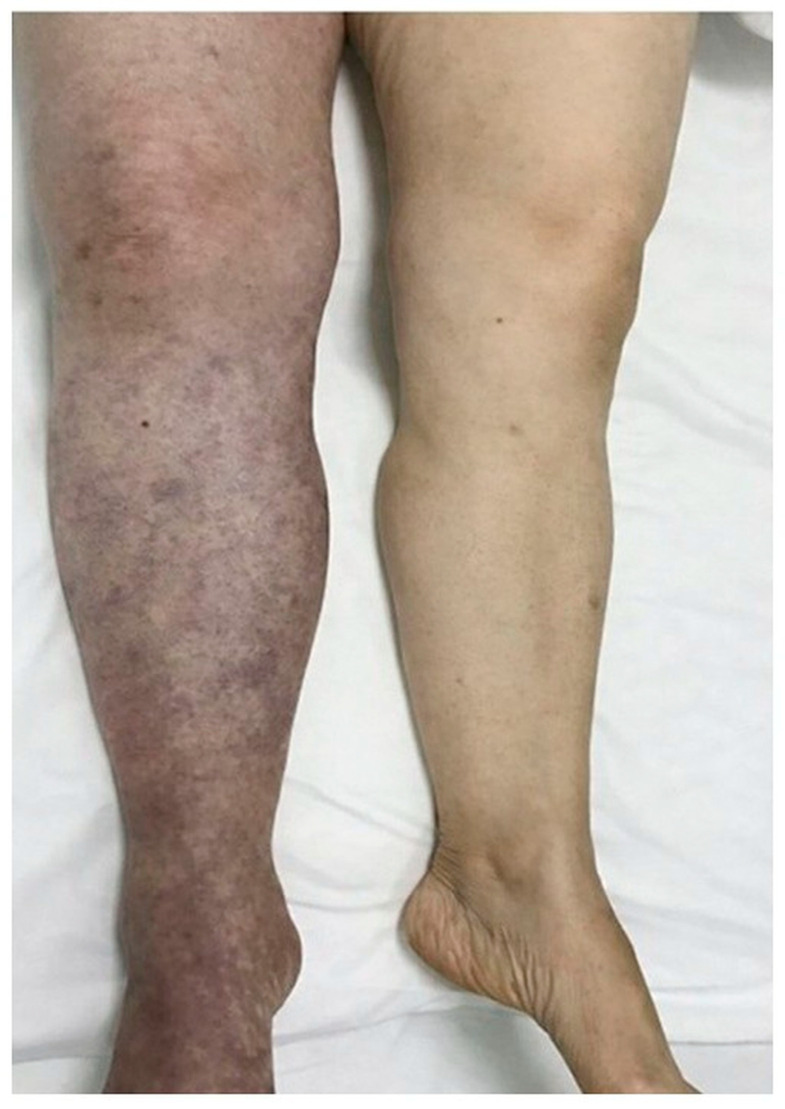
Representative clinical photograph illustrating advanced lower-extremity involvement in acute venous thromboembolism. Extensive limb edema with skin discoloration is shown, compatible with advanced venous outflow obstruction and a more severe clinical presentation.

Clinical presentation of acute venous thromboembolism demonstrated a spectrum of thromboinflammatory severity at baseline, ranging from isolated unilateral limb swelling with localized tenderness to extensive edema accompanied by erythema, warmth, and functional limitation consistent with advanced venous outflow obstruction. These clinical findings corresponded with baseline CSS distributions across the study cohort. Patients presenting with diffuse limb swelling and skin discoloration generally had higher baseline thromboinflammatory biomarker concentrations and longer recovery trajectories, whereas those with limited regional involvement tended to achieve earlier functional recovery. A representative clinical photograph illustrating advanced lower-extremity involvement is shown in Figure 3.

### 3.12. Molecular Docking Results

**Figure 4:**
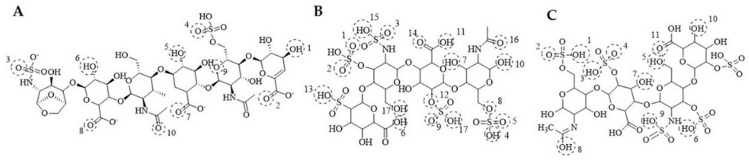
Representative 2D docking interaction maps of the evaluated low-molecular-weight heparins. (**A**) Enoxaparin. (**B**) Bemiparin. (**C**) Tinzaparin. Dashed circles indicate hydrogen-bond interaction regions, whereas numbers denote interacting atomic positions/residues.

Representative two-dimensional (2D) docking interaction maps for enoxaparin, bemiparin, and tinzaparin are shown in Figure 4. Quantitative docking outputs, including predicted binding energies, RMSD values, and hydrogen-bond interactions, are summarized in Supplementary Table S2.

**Figure 5:**
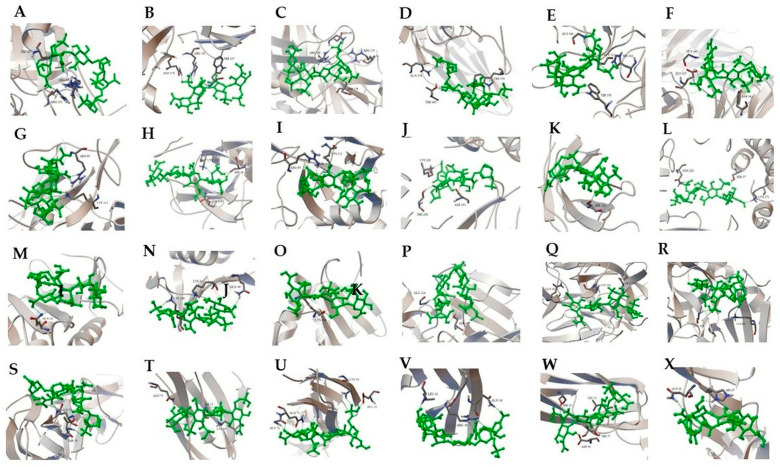
Three-dimensional docking conformations of enoxaparin, bemiparin, and tinzaparin with selected target proteins. (**A**) Enoxaparin–Thrombin (exosite II), (**B**) Bemiparin–Thrombin (exosite II), (**C**) Tinzaparin–Thrombin (exosite II), (**D**) Enoxaparin–Thrombin (exosite I), (**E**) Bemiparin–Thrombin (exosite I), (**F**) Tinzaparin–Thrombin (exosite I), (**G**) Enoxaparin–P-selectin, (**H**) Bemiparin–P-selectin, (**I**) Tinzaparin–P-selectin, (**J**) Enoxaparin–IL-6 receptor, (**K**) Bemiparin–IL-6 receptor, (**L**) Tinzaparin–IL-6 receptor, (**M**) Enoxaparin–PF4, (**N**) Bemiparin–PF4, (**O**) Tinzaparin–PF4, (**P**) Enoxaparin–Tissue factor, (**Q**) Bemiparin–Tissue factor, (**R**) Tinzaparin–Tissue factor, (**S**) Enoxaparin–ICAM-1, (**T**) Bemiparin–ICAM-1, (**U**) Tinzaparin–ICAM-1, (**V**) Enoxaparin–VCAM-1, (**W**) Bemiparin–VCAM-1, (**X**) Tinzaparin–VCAM-1. Ligand conformations and interacting residues within predicted binding regions are illustrated in each panel. Ligands are depicted in green, receptor protein structures in gray, and interaction contacts in blue.

The diagrams depict hydrogen-bond interactions and contact patterns between ligands and interacting residues. Three-dimensional (3D) ligand–protein docking conformations for thrombin exosite I and II and other selected targets are shown in Figure 5.

All ligands displayed plausible docking conformations within predicted binding regions. Owing to their large and flexible structures, LMWHs showed multiple potential contact points across extended binding surfaces. Docking poses were selected based on the lowest binding energy values and RMSD ≤ 2 Å. Detailed binding energies and interacting residues are provided in Supplementary Table S2, whereas docking parameters are detailed in Supplementary Section S9.

Interactions with adhesion molecules (ICAM-1, VCAM-1, and P-selectin), as well as IL-6 receptor, PF4, and tissue factor, are illustrated in Figure 5.

### 3.13. PPI Network Analysis

**Figure 6:**
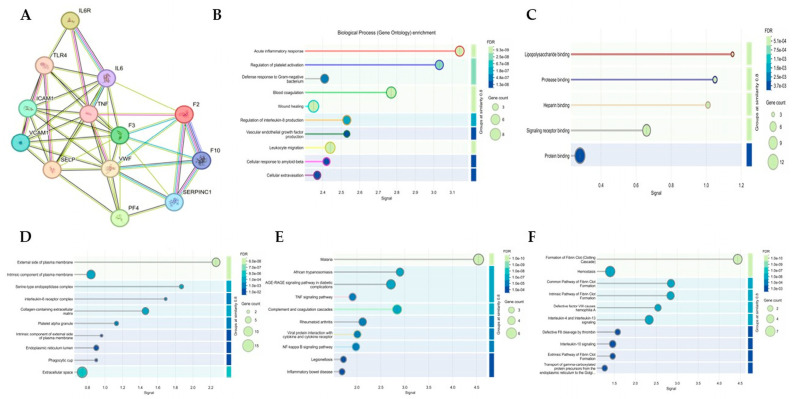
Integrated PPI network and functional enrichment analysis of docking-informed thromboinflammatory targets. (**A**) STRING network showing functional connectivity among coagulation factors, inflammatory mediators, and endothelial adhesion molecules. Colored lines represent different types of protein–protein interaction evidence provided by the STRING database (**B**) Gene Ontology biological process enrichment analysis. (**C**) Gene Ontology molecular function enrichment analysis. (**D**) Gene Ontology cellular component enrichment analysis. (**E**) Kyoto Encyclopedia of Genes and Genomes pathway enrichment analysis. (**F**) Reactome pathway enrichment analysis. Bubble size represents gene count, and color gradient corresponds to false discovery rate.

The PPI network derived from docking-informed targets is shown in Figure 6. Network topology parameters are summarized in Table 8. Enrichment analysis identified biological processes and pathways related to coagulation, inflammation, platelet activation, and endothelial interaction.

### 3.14. Hub Gene Identification

Hub genes were identified using the cytoHubba plugin. MCC analysis ranked F3, TNF, and IL6 as the top three hub genes (Table 9). Additional topological parameters, including degree, betweenness, and closeness centrality, also ranked these genes among the most connected nodes in the network (Table 10).

**Figure 7:**
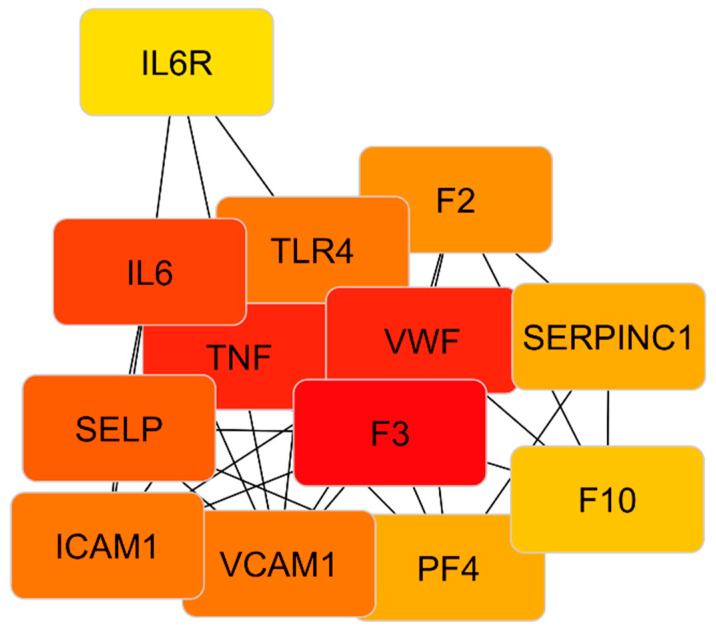
PPI network of thromboinflammatory targets constructed from docking-informed genes using STRING and visualized in Cytoscape. Nodes represent proteins, and edges indicate functional associations. Node color gradient from yellow to red reflects increasing degree centrality.

F3, TNF, IL6, and VWF showed high scores across multiple centrality measures, consistent with prominent topological positions within the interaction network (Figure 7).

Docking and network analyses identified an interaction framework centered on F3, TNF, IL6, and VWF, linking coagulation-related, inflammatory, and endothelial adhesion-associated targets. Concordant ranking of these genes across MCC and additional topological metrics was consistent with central positions within the network.

These observations are consistent with a multi-target pharmacological profile and provide systems-level context for the thromboinflammatory processes evaluated in this study.

Docking and network analyses collectively identified an integrated thromboinflammatory interaction framework centered on F3, TNF, IL6, and VWF, which represent convergence nodes linking coagulation activation, cytokine-mediated inflammation, and endothelial adhesion processes. Concordant identification of these genes across MCC and multiple centrality metrics was consistent with their prominent topological positions within the network. Together with molecular docking analyses suggesting predicted interactions of LMWHs with adhesion molecules, cytokine-related targets, and coagulation factors, these findings are consistent with a multi-target pharmacological profile extending beyond anticoagulation.

The observed network-level convergence was consistent with the clinical thromboinflammatory phenotype and may reflect coordinated modulation of thrombosis, inflammation, and endothelial activation.

### 3.15. Disease Enrichment Analysis

**Figure 8:**
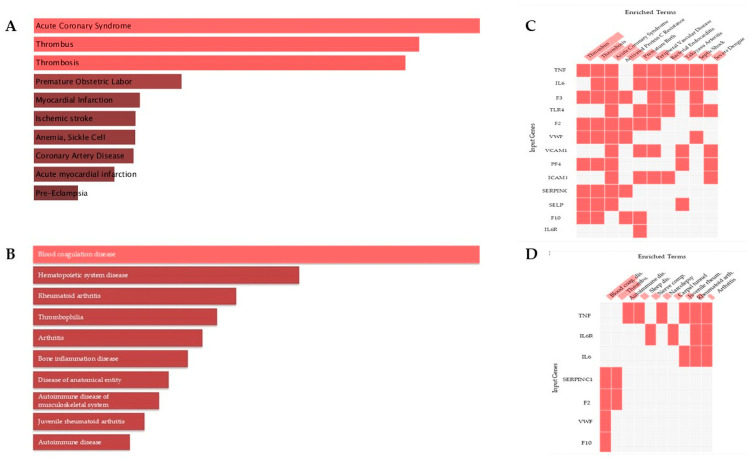
Disease enrichment analysis of docking-informed thromboinflammatory targets. (**A**) DisGeNET bar plot showing enriched thrombotic and cardiovascular disease terms. (**B**) Jensen DISEASES curated bar plot showing coagulation- and inflammation-related disease categories. (**C**) DisGeNET clustergram illustrating overlap between enriched disease terms and docking-informed targets. (**D**) Jensen DISEASES clustergram illustrating gene–disease term associations within enriched categories. Abbreviations: Blood coag. dis., blood coagulation disease; Thrombo., thromboembolic disease; Autoimmune dis., autoimmune disease; Sleep dis., sleep disorder; Nerve comp., nerve compression syndrome; Narcolepsy, narcolepsy; Carpal tunnel, carpal tunnel syndrome; Juvenile rheum., juvenile rheumatoid arthritis; Rheumatoid arth., rheumatoid arthritis.

Disease enrichment analysis identified associations between docking-informed targets and thromboinflammatory conditions. DisGeNET analysis highlighted acute coronary syndrome, thrombosis, thrombus formation, myocardial infarction, ischemic stroke, and coronary artery disease among the enriched terms. Jensen DISEASES analysis also identified blood coagulation disease, thrombophilia, and inflammatory rheumatologic conditions among enriched categories. These results were consistent with involvement of the identified targets in interconnected coagulation and inflammatory pathways relevant to venous thromboembolism (Figure 8; Table 11 and Table 12).

Additional functional enrichment analysis using the GO Biological Process library identified enrichment of inflammatory pathways, including regulation of interleukin-8 production, leukocyte migration, chemokine production, and cytokine-mediated signaling (Supplementary Figure S3; Supplementary Table S3). These results suggest involvement of the docking-informed targets in inflammatory and immune regulatory mechanisms.

Human Phenotype Ontology analysis identified clinically relevant thromboembolic phenotypes, including pulmonary embolism and deep venous thrombosis, as well as bleeding-related manifestations (Supplementary Figure S4; Supplementary Table S4). These findings are consistent with the clinical context of the thromboinflammatory targets.

## 4. Discussion

Acute venous thromboembolism represents a thromboinflammatory condition in which hemostatic and inflammatory pathways are functionally interconnected. In the present study, molecular docking analyses suggested that enoxaparin, bemiparin, and tinzaparin interact with multiple proteins involved in thrombin generation, inflammatory signaling, and endothelial adhesion. These predicted interactions are consistent with glycosaminoglycan binding domains enriched in positively charged residues that facilitate electrostatic interactions with sulfated polysaccharides such as heparin derivatives. Structural studies of thrombin exosite interactions support the presence of positively charged binding regions capable of accommodating glycosaminoglycan-like ligands, a concept that remains consistent with recent analyses of heparin–protein interactions in coagulation–inflammation coupling [11,24].

Similarly, crystallographic analyses of the IL-6 receptor complex have demonstrated electrostatically stabilized multi-subunit interfaces compatible with negatively charged ligands, a structural organization that remains consistent with recent analyses of glycosaminoglycan interactions with cytokines and related signaling proteins [13,25]. The interaction predicted at tissue factor–associated regions is consistent with structural descriptions of the factor VIIa–tissue factor complex, with exposed surfaces contributing to thrombin generation, in line with recent analyses highlighting factor VIIa-dependent signaling at the coagulation–inflammation interface [15,26].

These findings are consistent with the hypothesis that LMWHs may influence both coagulation-driven and immune-mediated amplification pathways and may shape coagulation–inflammation crosstalk involving tissue factor and downstream inflammatory mediators. Representative clinical findings illustrated in Figure 3 demonstrate the spectrum of clinical severity observed at presentation, ranging from unilateral edema to advanced limb discoloration and severe venous compromise. This variability supports the concept that venous thrombosis is not a static occlusive event but a dynamic process influenced by inflammatory amplification and microvascular dysfunction. Such dynamic behavior may, in selected cases, manifest as persistent thrombus propagation and collateral compromise despite appropriate anticoagulant therapy, as illustrated in Figure 1, which in selected cases may prompt consideration of catheter-directed interventions. The network pharmacology analysis is consistent with this interpretation, suggesting F3, TNF, IL6, and VWF as potential central nodes linking coagulation, inflammation, and endothelial activation. Tissue factor F3 emerged as a dominant hub, consistent with its upstream regulatory role in thrombin generation, while TNF and IL6 may represent inflammatory amplification loops. VWF links endothelial injury to platelet adhesion, further integrating thrombotic and inflammatory pathways. The interaction network suggested functional connectivity among these proteins, supporting a coherent coagulation–inflammation network.

The parallel reduction observed between inflammatory biomarkers and CSS is consistent with clinically measurable biological modulation. Early attenuation of hs-CRP and NLR during the initial treatment phase was associated with subsequent functional recovery trajectories, supporting the concept that early suppression of systemic inflammatory activity may represent an intermediate phenotype linking anticoagulant-associated biological effects with downstream clinical improvement. Attenuation of cytokine-driven endothelial activation may contribute to restoration of microvascular perfusion and promotion of endogenous fibrinolysis. Experimental studies have demonstrated that heparin derivatives can influence endothelial signaling and growth factor pathways, in line with pleiotropic vascular effects beyond anticoagulation and with recent investigations of heparin-mediated endothelial modulation [1]. In addition, network pharmacology analyses in venous thrombosis have identified overlapping inflammatory and coagulation pathways as potential therapeutic targets, in keeping with the present findings and recent systems-level analyses of integrated coagulation and inflammatory pathways in venous thrombosis [9,27]. An additional observation is the possibility of differential biological modulation among LMWHs. Although these agents share a common anticoagulant mechanism, structural heterogeneity and variable chain length may influence interactions with cytokines, adhesion molecules, and endothelial receptors. Such variability may partially explain differences in recovery kinetics observed clinically and may be consistent with pleiotropic pharmacodynamic effects within this drug class.

Importantly, alternative explanations for the observed differences in recovery trajectories should also be considered. Variability in dosing frequency, treatment adherence, and effective anticoagulant exposure may have contributed to the observed findings, particularly because anti-Xa activity, pharmacokinetic parameters, and injection adherence were not evaluated directly in this retrospective cohort. In addition, dose equivalence across LMWH preparations was based on institutional weight-adjusted prescribing protocols rather than pharmacokinetically verified exposure matching. Accordingly, the observed differences warrant cautious interpretation and should not be regarded as evidence of definitive clinical superiority among LMWH preparations.

The mediation analyses are consistent with the interpretation that early biomarker modulation may contribute to treatment-associated differences in recovery dynamics, while indicating that additional mechanisms beyond inflammatory attenuation are likely involved. Disease enrichment analyses further contextualized the identified targets by demonstrating associations with thromboembolic and inflammation-related conditions.

These enrichments support the biological plausibility of the docking-informed network and suggest that the identified proteins participate in shared coagulation–inflammation pathways rather than isolated signaling events. However, such associations should be interpreted with care, as enrichment reflects pathway overlap and does not establish direct causal relationships.

This study has several limitations. Molecular docking analyses were performed using static protein structures, which do not fully capture conformational flexibility under physiological conditions. In addition, docking algorithms and scoring functions may not accurately reflect binding affinities for large and highly flexible polyanionic molecules such as LMWHs. Protein–ligand interactions were evaluated under simplified solvent conditions, and potential contributions of water-mediated interactions and ionic microenvironment effects were not explicitly modeled. Furthermore, docking results represent computational predictions rather than direct evidence of biological activity and should be interpreted cautiously. The heterogeneous structure and variable chain lengths of LMWHs may also limit interpretation when docking is performed using representative conformers. Future studies incorporating molecular dynamics simulations and experimental validation are required to further clarify these interactions. Although IPTW-based covariate balance diagnostics demonstrated acceptable post-weighting balance and adequate propensity score overlap, residual confounding related to unmeasured variables cannot be excluded because of the retrospective observational study design. In addition, concomitant anticoagulant transitions, compression therapy utilization, and detailed renal function stratification were not uniformly standardized or systematically available across the retrospective cohort and may have contributed to residual clinical confounding. Longitudinal symptom trajectories were evaluated using a structured composite CSS developed for consistent follow-up assessment in the present study. The score enabled standardized longitudinal evaluation of symptom burden but does not represent a formally validated external clinical outcome instrument.

Despite these limitations, the present study integrates clinical recovery trajectories with longitudinal biomarker dynamics and docking-informed network pharmacology analyses, suggesting exploratory systems-level links between coagulation and inflammatory signaling; however, these findings remain hypothesis-generating and require experimental confirmation. Overall, the in silico results complement the clinical observations but do not establish direct mechanistic causality.

## 5. Conclusions

This retrospective study integrates clinical recovery trajectories with molecular docking, network pharmacology, and disease enrichment analyses to explore potential biological mechanisms associated with LMWHs in acute venous thromboembolism. The convergence of docking-informed targets and STRING-based network topology was consistent with coordinated coagulation, cytokine, and endothelial signaling interactions. Parallel improvement of CSS and inflammatory biomarkers during follow-up may reflect concurrent attenuation of coagulation–inflammation activity. Differences in recovery kinetics among LMWHs may reflect structural heterogeneity and pharmacodynamic variability within this drug class. These findings are hypothesis-generating. Prospective studies and experimental validation are required to confirm these mechanisms and clarify their clinical relevance.

## Figures and Tables

**Table 1:** Baseline demographic and clinical characteristics of patients treated with enoxaparin, bemiparin, and tinzaparin. Continuous variables are presented as mean ± standard deviation, and categorical variables as number (percentage).

Variable	Enoxaparin (*n* = 307)	Bemiparin (*n* = 304)	Tinzaparin (*n* = 310)	*p* Value
Age (years)	58.9 ± 13.2	59.4 ± 14.1	58.7 ± 13.6	0.82
Female sex	150 (48.9%)	155 (51.0%)	156 (50.3%)	0.88
BMI (kg/m^2^)	29.1 ± 4.8	29.4 ± 4.5	29.0 ± 4.7	0.63
Diabetes mellitus	86 (28.0%)	82 (27.0%)	90 (29.0%)	0.74
Hypertension	141 (46.0%)	137 (45.1%)	146 (47.1%)	0.81
Active smoking	104 (33.9%)	109 (35.9%)	108 (34.8%)	0.79
Baseline hs-CRP (mg/L)	11.8 ± 5.2	12.1 ± 5.5	11.6 ± 5.0	0.67
Proximal DVT	164 (53.4%)	163 (53.6%)	170 (54.8%)	0.93
Pulmonary embolism	86 (28.0%)	88 (28.9%)	84 (27.1%)	0.88
Previous PE history	21 (6.8%)	19 (6.2%)	23 (7.4%)	0.79
Previous contralateral DVT (>5 years)	26 (8.5%)	24 (7.9%)	29 (9.4%)	0.82
Thrombophilia (Factor V Leiden or homozygous MTHFR)	32 (10.4%)	30 (9.9%)	35 (11.3%)	0.86
Previous malignancy (remission)	33 (10.7%)	31 (10.2%)	35 (11.3%)	0.89
Postpartum DVT	9 (2.9%)	8 (2.6%)	10 (3.2%)	0.91

**Table 2:** Predisposing clinical conditions associated with the index venous thromboembolism. The distribution of predisposing factors is shown for each treatment group.

Clinical Variable	Enoxaparin	Bemiparin	Tinzaparin	Total (%)
Proximal DVT	164 (53.4%)	163 (53.6%)	170 (54.8%)	497 (54.0%)
Distal DVT	57 (18.6%)	53 (17.4%)	56 (18.1%)	166 (18.0%)
Pulmonary embolism	86 (28.0%)	88 (28.9%)	84 (27.1%)	258 (28.0%)
Postoperative thrombosis	69 (22.5%)	65 (21.4%)	70 (22.6%)	204 (22.1%)
Immobilization	82 (26.7%)	77 (25.3%)	82 (26.5%)	241 (26.2%)
Obesity-related VTE	57 (18.6%)	56 (18.4%)	60 (19.4%)	173 (18.8%)
Hormone therapy-related	21 (6.8%)	23 (7.6%)	22 (7.1%)	66 (7.2%)

Baseline thrombus localization characteristics are also summarized in Table 1.

**Table 3:** CSS system for symptom assessment in acute venous thromboembolism. The scoring system incorporates pain intensity, limb swelling, erythema and warmth, functional limitation, and systemic symptoms, with a total possible score ranging from 0 to 10.

Component	Clinical Description	Score Range
Pain Intensity	Patient-reported limb pain severity	0–4
Limb Swelling	Increase in limb circumference and sensation of tension	0–2
Erythema and Warmth	Local inflammatory signs over the affected area	0–1
Functional Limitation	Impaired ambulation or restriction of daily activities	0–2
Systemic Symptoms	Low-grade fever or general malaise	0–1
Total Possible Score	Sum of all components	0–10

Interpretation: 0 = complete recovery; 1–2 minimal symptoms; 3–5 mild–moderate; 6–8 marked; 9–10 severe. Assessment: Recorded at Days 0, 7, 20, 30, and 45. Definitions: ≥50% reduction = clinically meaningful improvement; CSS ≤ 2 = functional recovery.

**Table 4:** Time-to-event analysis of complete symptom resolution defined as CSS = 0 across treatment groups. Median recovery time, cumulative recovery probabilities, and inverse probability-weighted hazard ratios derived from Kaplan–Meier and Cox regression analyses are presented.

Outcome	Enoxaparin	Bemiparin	Tinzaparin
Median time to CSS = 0 (days)	31 (28–34)	28 (25–31)	24 (22–27)
30-day recovery probability	62%	71%	84%
45-day recovery probability	88%	93%	97%
Inverse probability-weighted hazard ratio vs. enoxaparin	Reference	1.28 (1.05–1.56)	1.72 (1.41–2.10)
Log-rank *p* value	—	0.014	<0.001

**Table 5:** Weighted clinical recovery endpoints and safety outcomes across treatment groups. Time to predefined CSS thresholds, analgesic requirements, recurrent symptoms during follow-up, and major bleeding events defined according to ISTH criteria are presented.

Clinical Endpoint	Enoxaparin	Bemiparin	Tinzaparin	*p* Value
Time to CSS ≤ 5 (days)	12.1 ± 3.6	10.3 ± 3.2	8.4 ± 2.9	<0.001
Time to CSS ≤ 2 (days)	24.3 ± 5.1	21.2 ± 4.7	17.6 ± 4.1	<0.001
Analgesic requirement > 10 days	102 (33.2%)	82 (27.0%)	61 (19.7%)	<0.001
Recurrent symptoms during follow-up	21 (6.8%)	16 (5.3%)	12 (3.9%)	0.18
Major bleeding (ISTH criteria)	4 (1.3%)	3 (1.0%)	3 (1.0%)	0.91

**Table 6:** Linear mixed-effects modeling of longitudinal thromboinflammatory biomarker trajectories. β coefficients represent time effects, treatment effects, and treatment-by-time interaction terms. Slope differences versus enoxaparin were used to estimate relative rates of biomarker change during follow-up.

Biomarker	Time Effect β	Treatment Effect β	Treatment × Time Interaction β	Slope Difference vs. Enoxaparin (Bemiparin)	Slope Difference vs. Enoxaparin (Tinzaparin)	*p* Interaction
hs-CRP	−0.48	−0.21	−0.34	−0.12	−0.34	<0.001
MPV	−0.31	−0.14	−0.29	−0.09	−0.29	<0.001
NLR	−0.39	−0.17	−0.36	−0.13	−0.36	<0.001
PLR	−0.24	−0.11	−0.26	−0.08	−0.26	0.002
ESR	−0.22	−0.09	−0.23	−0.07	−0.23	0.006
Fibrinogen	−0.17	−0.06	−0.19	−0.05	−0.19	0.004

**Table 7:** Early reductions in thromboinflammatory biomarkers and their associations with functional recovery trajectories. Percent reductions in hs-CRP, MPV, NLR, and fibrinogen between baseline and Day 7 are shown together with inverse probability-weighted Cox regression estimates for time to functional recovery defined as CSS ≤ 2.

Metric	Enoxaparin	Bemiparin	Tinzaparin	HR per 10% Reduction (95% CI)	Proportion Mediated (%)	*p* Value
hs-CRP	32%	37%	46%	1.18 (1.09–1.27)	34%	<0.001
MPV	29%	34%	41%	1.11 (1.03–1.19)	19%	0.006
NLR	26%	29%	38%	1.14 (1.06–1.22)	27%	0.002
Fibrinogen	18%	21%	28%	1.07 (0.99–1.15)	11%	0.081

**Table 8:** Top enriched Gene Ontology biological processes and Kyoto Encyclopedia of Genes and Genomes pathways identified from STRING enrichment analysis.

Category	Term/Pathway	Gene Count	FDR	Matching Genes
GO Biological Process	Acute inflammatory response	6	9.29 × 10^−8^	VCAM1, F2, F3, IL6R, IL6, TNF
GO Biological Process	Blood coagulation	7	9.29 × 10^−8^	VWF, PF4, F2, F3, SERPINC1, F10, IL6
GO Biological Process	Wound healing	8	9.29 × 10^−8^	VWF, PF4, F2, F3, SERPINC1, TLR4, F10, IL6
GO Biological Process	Leukocyte migration	7	1.46 × 10^−7^	SELP, ICAM1, VCAM1, PF4, IL6R, IL6, TNF
GO Biological Process	Regulation of platelet activation	5	3.05 × 10^−7^	SELP, F2, IL6R, TLR4, IL6
GO Biological Process	Regulation of interleukin-8 production	5	1.94 × 10^−6^	F3, IL6R, TLR4, IL6, TNF
GO Biological Process	Cellular extravasation	4	1.12 × 10^−5^	SELP, ICAM1, VCAM1, TNF
GO Biological Process	Vascular endothelial growth factor production	3	1.32 × 10^−5^	IL6R, IL6, TNF
KEGG pathway	Complement and coagulation cascades	5	3.13 × 10^−7^	VWF, F2, F3, SERPINC1, F10
KEGG pathway	AGE-RAGE signaling pathway in diabetic complications	5	4.46 × 10^−7^	ICAM1, VCAM1, F3, IL6, TNF
KEGG pathway	Viral protein interaction with cytokine and cytokine receptor	4	2.41 × 10^−5^	PF4, IL6R, IL6, TNF
KEGG pathway	NF-kappa B signaling pathway	4	2.51 × 10^−5^	ICAM1, VCAM1, TLR4, TNF
KEGG pathway	TNF signaling pathway	4	3.17 × 10^−5^	ICAM1, VCAM1, IL6, TNF

**Table 9:** Hub gene ranking based on MCC using the cytoHubba plugin in Cytoscape.

Rank	Gene	MCC Score
1	F3	1518
2	TNF	1494
3	IL6	1470
4	SELP	1464
5	ICAM1	1440
6	VCAM1	1440
7	VWF	798
8	TLR4	726
9	F2	48
10	PF4	30
11	SERPINC1	30
12	F10	24
13	IL6R	6

**Table 10:** Topological parameters of hub genes identified by cytoHubba, including degree, betweenness, and closeness centrality.

Gene	Degree	Betweenness	Closeness
F3	11	19.607	11.5
TNF	10	13.690	11.0
VWF	10	13.404	11.0
IL6	9	8.774	10.5
SELP	8	2.500	10.0
ICAM1	7	0.333	9.5
VCAM1	7	0.333	9.5
TLR4	7	3.202	9.5
F2	6	3.571	9.0
PF4	5	1.417	8.5
SERPINC1	5	1.167	8.33
F10	4	0	7.83
IL6R	3	0	7.17

**Table 11:** Top enriched diseases identified by DisGeNET analysis of docking-informed thromboinflammatory targets ranked by adjusted *p*-value.

Rank	Disease	Adjusted *p*-Value	Odds Ratio	Combined Score
1	Acute Coronary Syndrome	2.12 × 10^−16^	445.03	19,522.04
2	Thrombus	6.27 × 10^−16^	839.96	35,354.97
3	Thrombosis	6.27 × 10^−16^	503.04	20,970.67
4	Premature Obstetric Labor	3.31 × 10^−13^	328.08	11,525.60
5	Myocardial Infarction	6.76 × 10^−13^	239.41	8118.31
6	Ischemic stroke	6.76 × 10^−13^	170.16	5748.45
7	Anemia, Sickle Cell	6.76 × 10^−13^	200.32	6765.65
8	Coronary Artery Disease	6.76 × 10^−13^	235.52	7942.72
9	Acute myocardial infarction	1.05 × 10^−12^	186.70	6191.79
10	Pre-Eclampsia	2.76 × 10^−12^	220.48	7076.30

**Table 12:** Top enriched disease categories identified by Jensen DISEASES curated analysis of docking-informed thromboinflammatory targets.

Rank	Disease	Adjusted *p*-Value	Odds Ratio	Genes
1	Blood Coagulation Disease	1.39 × 10^−7^	354.88	F10, VWF, SERPINC1, F2
2	Hematopoietic System Disease	9.33 × 10^−5^	53.07	F10, VWF, SERPINC1, F2
3	Rheumatoid Arthritis	2.19 × 10^−4^	87.88	IL6, TNF, IL6R
4	Thrombophilia	2.41 × 10^−4^	454.07	SERPINC1, F2
5	Arthritis	2.51 × 10^−4^	70.24	IL6, TNF, IL6R
6	Bone Inflammation Disease	2.98 × 10^−4^	62.16	IL6, TNF, IL6R
7	Disease of Anatomical Entity	6.27 × 10^−4^	10.16	IL6, F10, VWF, SERPINC1, F2, TNF, IL6R, TLR4
8	Autoimmune Disease of Musculoskeletal System	6.27 × 10^−4^	43.47	IL6, TNF, IL6R
9	Juvenile Rheumatoid Arthritis	8.91 × 10^−4^	139.59	IL6, TNF
10	Autoimmune Disease	8.91 × 10^−4^	35.60	IL6, TNF, IL6R

## Data Availability

The original contributions presented in this study are included in the article/Supplementary Material. Further inquiries can be directed to the corresponding author.

## Supplementary Materials

The following supporting information can be downloaded at: https://www.mdpi.com/article/10.3390/medsci14020260/s1, Figure S1: Additional fluoroscopic examples of venous obstruction, collateral filling, and thrombus burden in medically less responsive lower-extremity deep vein thrombosis; Figure S2: Propensity score diagnostics and covariate balance assessment after inverse probability of treatment weighting; Figure S3: GO Biological Process enrichment analysis of docking-derived thromboinflammatory targets; Figure S4: Human Phenotype Ontology enrichment analysis of docking-derived thromboinflammatory targets; Table S1: Standardized mean differences before and after inverse probability of treatment weighting; Table S2: Molecular docking interactions and binding energies; Table S3: GO Biological Process enrichment analysis of docking-derived thromboinflammatory targets; Table S4: Human Phenotype Ontology enrichment analysis of docking-derived thromboinflammatory targets.

## Abbreviations

The following abbreviations are used in this manuscript:

BP	Biological process
CC	Cellular component
CSS	Clinical Severity Score
DVT	Deep vein thrombosis
GO	Gene Ontology
ICAM-1	Intercellular adhesion molecule-1
IL-6R	Interleukin-6 receptor
KEGG	Kyoto Encyclopedia of Genes and Genomes
LMWH	Low-molecular-weight heparin
MF	Molecular function
MPV	Mean platelet volume
NLR	Neutrophil-to-lymphocyte ratio
PF4	Platelet factor 4
PPI	Protein–protein interaction
RMSD	Root-mean-square deviation
STRING	Search Tool for the Retrieval of Interacting Genes/Proteins
VCAM-1	Vascular cell adhesion molecule-1
VTE	Venous thromboembolism
hs-CRP	High-sensitivity C-reactive protein
