# Supplementary material for: Thromboinflammatory and Pharmacological Effects of Low-Molecular-Weight Heparins in Acute Venous Thromboembolism: An Integrated Clinical and In Silico Analysis
Source: Med Sci (Basel). 2026 May 19;14(2):260. doi: 10.3390/medsci14020260 (PMC13214951; doi:10.3390/medsci14020260)
Supplement: Supplementary file 1 [file medsci-14-00260-s001.zip › medsci-4265620-supplementary.pdf]

Supplementary Materials

Thromboinflammatory and Pharmacological Effects of Low-Molecular-Weight Heparins in Acute Venous Thrombo-embolism: An Integrated Clinical and *In Silico* Analysis

This supplementary document provides extended methodological details supporting the analyses presented in the main manuscript. To improve readability of the main text, detailed eligibility criteria, expanded statistical modeling procedures, and technical aspects of the in silico computational workflow, including docking, network, and enrichment analyses, are presented in this supplementary file.

S1. Study Population and Eligibility Criteria

Detailed inclusion and exclusion criteria applied in the present retrospective cohort study are provided below to ensure transparency and reproducibility.

Inclusion Criteria

Patients were eligible for inclusion if they were 18 years of age or older and had objectively confirmed acute venous thromboembolism. Diagnosis was established using compression ultrasonography, computed tomography pulmonary angiography, or other standard imaging modalities according to institutional clinical practice. Only patients who received therapeutic-dose enoxaparin, bemiparin, or tinzaparin as initial anticoagulant therapy at hospital admission were included in the study cohort.

Exclusion Criteria

Exclusion criteria included active malignancy at the time of diagnosis, pregnancy, known thrombophilia requiring alternative anticoagulation strategies, prior anticoagulant therapy before admission, chronic inflammatory or autoimmune disease, severe hepatic or renal insufficiency, recent major surgery or trauma within the previous four weeks, ongoing infection, hematologic disorders affecting platelet count, and incomplete clinical or laboratory follow-up data. Patients with a previous history of malignancy in remission were not excluded.

Patients receiving systemic thrombolytic therapy or requiring surgical thrombectomy at presentation were also excluded in order to minimize potential confounding effects on thrombus resolution and inflammatory biomarker trajectories.

Additional Exclusion Criteria for Longitudinal Analysis

Patients were excluded from the final analytical cohort if they had contraindications to LMWH therapy, treatment discontinuation at baseline, incomplete laboratory measurements, incomplete follow-up clinical assessments at predefined evaluation time points, ongoing infection, chronic inflammatory disease, autoimmune disorders, recent major surgery or trauma, or hematologic conditions potentially affecting inflammatory biomarker interpretation.

S2. Clinical Variables and Data Definitions

The following baseline demographic characteristics and clinical variables were recorded from electronic medical records and predefined data collection forms.

Demographic Variables

Age, sex, and body mass index were recorded at baseline.

Clinical Risk Factors

Clinical risk factors included diabetes mellitus, hypertension, smoking status, prior history of pulmonary embolism, previous contralateral deep vein thrombosis older than five years, previous history of malignancy in remission, postpartum status, and inherited thrombophilia markers including Factor V Leiden mutation and homozygous MTHFR gene mutation.

Thromboembolic Presentation

Thromboembolic presentation was categorized as proximal deep vein thrombosis, distal deep vein thrombosis, pulmonary embolism, or combined involvement.

Treatment and Timing Variables

Treatment allocation and timing of therapy initiation were extracted from archived clinical documentation.

Follow-up Assessments

Laboratory measurements and clinical severity assessments were recorded at predefined follow-up time points using structured outpatient visit documentation.

S3. Treatment Protocol and Adjunctive Management

Treatment indications included proximal or distal deep vein thrombosis, pulmonary embolism, postoperative venous thrombosis, thrombosis associated with previous malignancy in remission, prolonged immobilization, hormonal exposure, and postpartum venous thrombosis.

Patients received weight-adjusted therapeutic dosing of enoxaparin, bemiparin, or tinzaparin according to institutional protocols. Dose adjustments were performed when clinically indicated based on renal function, body weight, and bleeding risk. Treatment adherence and continuity were verified through prescription records and structured follow-up documentation.

Patients with insufficient clinical improvement during early follow-up were evaluated for adjunctive interventional treatment. In selected patients with persistent symptom burden and radiologically significant thrombus load, catheter-directed thrombolysis was performed following multidisciplinary decision making. These patients were retained in the overall cohort and evaluated separately in exploratory subgroup analyses.

In patients considered at increased recurrence risk, inferior vena cava filter placement was performed when clinically indicated. Duration of filter placement was determined individually according to thrombotic risk profile and follow-up imaging findings.

S4. Clinical Severity Score and Outcome Definitions

The Clinical Severity Score ranged from 0 to 10 and incorporated pain intensity, limb swelling, erythema, warmth, and functional limitation using predefined ordinal grading criteria. Each component contributed proportionally to the composite score to enable reproducible longitudinal assessment.

Clinical Severity Score measurements were obtained at each predefined follow-up visit. Recovery trajectories were evaluated based on time-dependent score changes. Functional recovery was defined as a sustained Clinical Severity Score ≤2, and complete recovery as a score of 0.

Temporal relationships between thromboinflammatory biomarker reductions and clinical improvement were assessed to explore potential associations between biomarker trajectories and recovery dynamics.

The CSS was used as a structured longitudinal clinical assessment tool designed to provide consistent evaluation of symptom burden during follow-up. The score was not intended to represent a formally validated external clinical scale, and findings derived from CSS trajectories should therefore be interpreted in conjunction with objective thromboinflammatory biomarkers and other supportive clinical outcomes.

S5. Laboratory Measurements and Quality Control

Laboratory biomarkers were selected to represent platelet activation, systemic inflammatory response, and thromboinflammatory activity associated with venous thromboembolism. All laboratory analyses were performed in the central institutional laboratory using standardized automated hematology analyzers operating under routine internal and external quality control procedures. Sample collection and processing followed institutional laboratory protocols to ensure measurement consistency across time points.

S6. Statistical Modeling Procedures

Propensity Score Weighting

To address potential baseline imbalances arising from nonrandom treatment allocation, inverse probability of treatment weighting based on propensity scores was applied. Propensity scores representing the probability of receiving each low-molecular-weight heparin preparation were estimated using multinomial logistic regression models. Covariates included demographic characteristics, clinical risk factors, thromboembolic presentation variables, baseline inflammatory status, and inherited thrombophilia markers. Stabilized inverse probability weights were calculated and incorporated into outcome models to improve comparability between treatment groups. Covariate balance after weighting was evaluated using standardized mean differences, with values below 0.10 considered indicative of adequate balance.

Covariate balance and propensity score overlap were additionally evaluated after weighting using standardized mean differences and visual inspection of weighted propensity score distributions across treatment groups. The weighted distributions demonstrated adequate overlap without evidence of

substantial positivity violation. Detailed propensity score diagnostics are presented in Supplementary Figure S1, and numerical standardized mean difference values before and after weighting are summarized in Supplementary Table S1.

Table S1. Absolute standardized mean differences before and after inverse probability of treatment weighting (IPTW). Covariate balance was assessed for all prespecified baseline variables included in the multinomial propensity score model. Post-weighting values below 0.10 were considered indicative of acceptable balance.

Covariate	SMD Before IPTW	SMD After IPTW
Age	0.037	0.018
Female sex	0.024	0.011
BMI	0.041	0.022
Diabetes mellitus	0.028	0.014
Hypertension	0.031	0.016
Active smoking	0.033	0.019
Baseline hs-CRP	0.045	0.021
Proximal DVT	0.018	0.009
Pulmonary embolism	0.027	0.013
Previous PE history	0.039	0.017
Previous contralateral DVT (>5 years)	0.044	0.020
Thrombophilia (FVL/homozygous MTHFR)	0.042	0.019
Previous malignancy in remission	0.033	0.015
Postpartum DVT	0.018	0.008

Abbreviations: SMD, standardized mean difference; IPTW, inverse probability of treatment weighting; DVT, deep vein thrombosis; PE, pulmonary embolism; FVL, Factor V Leiden; MTHFR, methylenetetrahydrofolate reductase; hs-CRP, high-sensitivity C-reactive protein; BMI, body mass index. All post-weighting SMD values were below the prespecified 0.10 threshold, supporting acceptable covariate balance across treatment groups.

Longitudinal Biomarker Modeling

Longitudinal changes in thromboinflammatory biomarkers were analyzed using linear mixed-effects regression models. Fixed effects included treatment group, follow-up time point, and treatment-by-time interaction terms. Random intercept and random slope terms for time were specified at the patient level to account for baseline variability and interindividual differences in biomarker change rates.

Follow-up time was modeled as a categorical variable corresponding to baseline and Days 7, 20, and 45 assessments. An unstructured covariance matrix was specified to allow flexible estimation of within-patient correlations. Sensitivity analyses using alternative covariance structures, including first-order autoregressive correlation, were performed to confirm robustness of trajectory estimates.

Inverse probability of treatment weighting was incorporated into mixed-effects models. Models were additionally adjusted for prespecified baseline covariates. Biomarker variables with skewed distributions were analyzed using log-transformed values when appropriate. Model adequacy was evaluated using residual diagnostics, inspection of variance components, and likelihood-based model comparison procedures.

These models enabled evaluation of treatment-associated trajectories of thromboinflammatory biomarkers across follow-up assessments while accounting for clinically relevant heterogeneity.

S7. Mediation Analysis

Causal mediation analyses were performed to investigate whether treatment-associated differences in clinical recovery trajectories were partially explained by early changes in thromboinflammatory biomarkers. Early biomarker changes between baseline and Day 7 were evaluated as candidate mediators linking treatment allocation to time to functional recovery defined as achievement of a Clinical Severity Score of 2 or lower.

Separate mediation models were constructed for high-sensitivity C-reactive protein, neutrophil-to-lymphocyte ratio, mean platelet volume, and fibrinogen to evaluate their individual contributions to recovery dynamics. Natural direct and indirect effects were estimated to quantify the proportion of treatment-associated differences explained by early attenuation of thromboinflammatory activity.

Models incorporated inverse probability of treatment weighting and were adjusted for prespecified baseline covariates. Time to functional recovery was modeled using survival-based mediation procedures. Bias-corrected confidence intervals for indirect effects were obtained using nonparametric

bootstrap resampling with 5000 iterations. The proportion mediated was calculated to estimate the relative contribution of early biomarker modulation to treatment-associated recovery differences.

All mediation analyses were performed within the R statistical environment using regression-based causal mediation procedures.

S8. Early Biomarker Response and Subgroup Analyses

Early biomarker response was evaluated by quantifying relative changes in thromboinflammatory markers between baseline and Day 7. Relative change values were calculated for high-sensitivity C-reactive protein, neutrophil-to-lymphocyte ratio, mean platelet volume, platelet-to-lymphocyte ratio, erythrocyte sedimentation rate, and fibrinogen concentrations. Patients were stratified according to magnitude of early biomarker reduction using tertile-based categorization.

Associations between early biomarker response and subsequent clinical improvement were evaluated using inverse probability–weighted Cox proportional hazards regression models with time to functional recovery as the primary outcome. Models were adjusted for prespecified baseline covariates. Interaction analyses were performed according to thrombus localization, presence of pulmonary embolism, inherited thrombophilia status, and postpartum venous thrombosis. Kaplan–Meier analyses stratified by tertiles of early biomarker reduction were constructed to visualize differences in recovery trajectories.

Prespecified subgroup analyses were conducted according to thrombus localization, presence of concomitant pulmonary embolism, baseline inflammatory burden, and inherited thrombophilia. Additional subgroup and sensitivity analyses were performed according to postpartum venous thrombosis, prior contralateral deep vein thrombosis, sex, smoking status, and previous malignancy in remission.

S9. Molecular Docking Workflow

S9.1. Software Environment

Molecular docking simulations and preprocessing steps were performed using AutoDock 4.2.6 (The Scripps Research Institute, La Jolla, CA, USA). Ligand and receptor preparation was conducted using AutoDockTools (MGLTools) version 1.5.6. File format conversion and structure processing were performed using Open Babel version 3.1.1. Visualization and structural inspection of docking poses

were carried out using PyMOL version 2.5 (Schrödinger, LLC, New York, NY, USA) and Python Molecular Viewer (PMV) version 1.5.6. All computations were performed under identical software configurations to maintain consistency across docking experiments.

S9.2. Ligand Preparation

Ligand structures for enoxaparin, bemiparin, and tinzaparin were generated using validated chemical representations and converted into three-dimensional conformations. Geometry optimization was performed prior to docking using energy minimization procedures. Polar hydrogen atoms were added, and Gasteiger partial charges were assigned using AutoDockTools. Rotatable bonds were defined according to AutoDock conventions, and ligand structures were saved in PDBQT format. Due to the flexible and polyanionic nature of low-molecular-weight heparins, conformations with minimized steric clashes were selected for docking analyses.

S9.3. Receptor Preparation

Protein structures were retrieved from the Protein Data Bank. Receptor preprocessing included removal of crystallographic water molecules, addition of polar hydrogen atoms, assignment of Kollman charges, and merging of nonpolar hydrogens. Each receptor was saved in PDBQT format. Docking grids were centered on biologically relevant binding regions based on structural information provided in the original crystallographic studies. Grid parameters were kept consistent across ligand comparisons for each receptor.

S9.4. Target-Specific Docking Parameters

Docking simulations were performed using the Lamarckian genetic algorithm implemented in AutoDock 4.2.6. Grid spacing was set to 0.375 Å for all targets. Ten independent docking runs were performed for each ligand–receptor pair. The grid box center and size were defined individually for each target protein as follows:

Thrombin exosite I (PDB ID: 5E8E): grid center (x = −15.2, y = 24.6, z = 8.3); grid size (60 × 60 × 60)

Thrombin exosite II (PDB ID: 3B9F): grid center (x = −10.4, y = 18.1, z = 12.7); grid size (60 × 60 × 60)

P-selectin (PDB ID: 1G1S): grid center (x = 5.2, y = −14.8, z = 22.6); grid size (60 × 60 × 60)

IL-6 receptor (PDB ID: 1P9M): grid center (x = 10.7, y = −3.9, z = 15.4); grid size (60 × 60 × 60)

Platelet factor 4 (PDB ID: 4R9W): grid center (x = −7.3, y = 9.5, z = 13.1); grid size (60 × 60 × 60)

Tissue factor (PDB ID: 1DAN): grid center (x = 12.4, y = −6.2, z = 19.8); grid size (60 × 60 × 60)

ICAM-1 (PDB ID: 1IC1): grid center (x = 3.5, y = 8.1, z = −11.4); grid size (60 × 60 × 60)

VCAM-1 (PDB ID: 1IJ9): grid center (x = −6.8, y = 14.3, z = 4.6); grid size (60 × 60 × 60)

S9.5. Pose Selection Criteria

Docking poses were evaluated according to predicted binding energy, root-mean-square deviation (RMSD), and presence of hydrogen-bond interactions within functionally relevant binding regions. Conformations with RMSD ≤ 2 Å were considered stable docking solutions. For each ligand–receptor pair, the pose with the lowest binding energy that satisfied stability criteria was selected for downstream analysis. Due to the flexible polysaccharide structure of low-molecular-weight heparins, docking results should be interpreted cautiously. Binding energy differences were not considered quantitative affinity estimates but rather indicators of relative interaction stability across docking poses.

Detailed molecular docking results, functional enrichment outputs, and disease association analyses are provided in the following Supplementary Tables. Supplementary Table S2 summarizes binding energies and hydrogen-bond interactions derived from docking analyses. Supplementary Tables S3 and S4 present Gene Ontology biological process enrichment and Human Phenotype Ontology enrichment results, respectively.

Comparisons between ligands were performed qualitatively, and no ranking of binding affinity was inferred from docking energies due to structural heterogeneity of the ligands.

Table S2. Binding energies, RMSD values, and key hydrogen-bond interactions of enoxaparin, bemiparin, and tinzaparin with selected protein targets.

	Binding Energy (kcal/mol)	RMSD	H-bond interactions (distance, Å)	PDB ID
Enoxaparin	-16.43	0.00	Enoxaparin O-1 with H-1 of Trp237 (2.238); Enoxaparin O-2 with H-1 of Arg101 (1.996); Enoxaparin O-3 with H-1 of	3B9F

			Arg93 (1.787); Enoxaparin O-4 with H-1 of Gln244 (1.842)	
Bemiparin	-10.36	0.02	Bemiparin O-1 with H-1 of Arg101 (1.985); Bemiparin O-1 with H-2 of Arg101 (2.198); Bemiparin O-2 with H-1 of Asn179 (1.784); Bemiparin O-3 with H-1 of Trp237 (2.159)	3B9F
Tinzaparin	-9.00	0.00	Tinzaparin O-1 with H-1 of Asn95 (1.968); Tinzaparin O-2 with H-1 of Arg101 (1.647); Tinzaparin O-1 with H-1 of Arg175 (2.170); Tinzaparin O-3 with H-1 of Asp178 (1.889)	3B9F
Enoxaparin	-10.71	0.00	Enoxaparin O-5 with H-2 of Trp370 (1.678); Enoxaparin O-3 with H-1 of Gln476 (2.048); Enoxaparin H-6 with O-1 of Thr467 (2.108)	5E8E
Bemiparin	-9.69	0.00	Bemiparin O-4 with H-1 of His363 (2.118); Bemiparin O-6 with H-2 of Trp370 (1.923); Bemiparin O-5 with H-1 of Gly523 (1.868); Bemiparin O-7 with H-1 of Gly548 (2.153)	5E8E
Tinzaparin	-8.53	0.00	Tinzaparin O-4 with H-1 of Tyr367 (1.979); Tinzaparin O-5 with H-1 of Gly550 (1.954); Tinzaparin H-6 with O-1 of Glu522 (2.233)	5E8E
Enoxaparin	-6.27	0.00	Enoxaparin O-8 with H-1 of Arg85 (1.929); Enoxaparin O-8 with H-2 of Arg85 (1.983); Enoxaparin O-2 with H-2 of Arg85 (1.983); Enoxaparin O-7 with H-1 of Lys111 (1.873); Enoxaparin O-7 with H-1 of Lys111 (2.034)	1G1S
Bemiparin	-9.50	0.00	Bemiparin O-8 with H-1 of Lys111 (2.073); Bemiparin O-9 with H-1 of Asn105 (1.827); Bemiparin H-4 with O-1 of Ser46 (1.957); Bemiparin H-10 with O-1 of Ser46 (1.956)	1G1S
Tinzaparin	-8.46	1.64	Tinzaparin O-7 with H-1 of Arg85 (1.747); Tinzaparin O-4 with H-1 of Arg85 (2.130); Tinzaparin O-3 with H-2 of Arg85 (1.633); Tinzaparin O-4 with H-1 of Lys111 (2.195)	1G1S
Enoxaparin	-13.61	0.00	Enoxaparin O-2 with H-1 of Lys228 (1.980); Enoxaparin O-7 with H-1 of Thr258 (1.980); Enoxaparin H-6 with O-1 of Asp253 (1.980)	1P9M

Bemiparin	-11.72	0.00	Bemiparin O-11 with H-1 of Ser229 (2.643)	1P9M
Tinzaparin	-10.16	0.00	Tinzaparin O-6 with H-1 of Asn224 (1.803); Tinzaparin O-3 with H-1 of Ser37 (1.973); Tinzaparin O-3 with H-1 of Lys171 (2.244)	1P9M
Enoxaparin	-5.28	0.00	Enoxaparin O-7 with H-1 of Gln40 (2.896)	4R9W
Bemiparin	-4.94	0.00	Bemiparin O-12 with H-1 of Lys31 (2.203); Bemiparin O-6 with H-2 of Lys31 (2.124); Bemiparin O-13 with H-2 of Gln40 (2.092); Bemiparin O-5 with H-1 of Lys146 (1.817)	4R9W
Tinzaparin	-4.24	0.00	Tinzaparin H-5 with O-1 of Lys146 (2.239)	4R9W
Enoxaparin	-9.80	0.00	Enoxaparin O-9 with H-1 of Gly216 (1.590); Enoxaparin H-1 with O-1 of Ser195 (2.076)	1DAN
Bemiparin	-10.98	0.00	Bemiparin O-14 with H-1 of Lys192 (2.019); Bemiparin O-15 with H-1 of Gly219 (1.921); Bemiparin H-10 with H-1 of Gln40 (1.836)	1DAN
Tinzaparin	-8.79	0.00	Tinzaparin O-4 with H-1 of Lys60 (1.866); Tinzaparin O-9 with H-1 of Gly193 (2.112)	1DAN
Enoxaparin	-5.41	0.00	Enoxaparin O-7 with H-1 of Gln73 (2.205); Enoxaparin O-3 with H-1 of Thr75 (2.206)	1IC1
Bemiparin	-5.36	0.00	Bemiparin O-17 with H-1 of Ile33 (1.623); Bemiparin O-16 with H-2 of Lys39 (1.963); Bemiparin O-10 with H-1 of Lys39 (1.891); Bemiparin O-12 with H-1 of Lys39 (1.847); Bemiparin O-13 with H-1 of Gln73 (2.149)	1IC1
Tinzaparin	-7.68	0.00	Tinzaparin O-2 with H-1 of Lys50 (2.172); Tinzaparin H-1 with O-1 of Glu41 (1.896); Tinzaparin O-10 with H-1 of Gly72 (2.193); Tinzaparin H-10 with O-1 of Gly72 (1.916); Tinzaparin O-6 with H-1 of Gln73 (2.147)	1IC1
Enoxaparin	-5.66	0.00	Enoxaparin O-1 with H-1 of Arg36 (2.207); Enoxaparin O-10 with NH-1 of Arg36 (2.085); Enoxaparin O-2 with H-1 of Gln38 (1.930); Enoxaparin O-6 with H-1 of Leu43 (1.958)	1IJ9

Bemiparin	-6.11	0.00	Bemiparin O-8 with H-1 of Ser34 (2.249); Bemiparin O-8 with H-1 of Thr37 (2.206); Bemiparin O-17 with H-1 of Leu43 (1.987); Bemiparin H-7 with O-1 of Asp40 (2.039)	1IJ9
Tinzaparin	-4.20	0.00	Tinzaparin O-11 with H-1 of Gln38 (2.161); Tinzaparin O-5 with H-1 of His67 (2.031)	1IJ9

Table S3. GO Biological Process enrichment analysis of docking-derived thromboinflammatory targets. Enriched biological processes were identified using the Enrichr platform. Terms are ranked according to adjusted p-values. Overlap indicates the number of genes associated with each biological process.

Term	Overlap	Adjusted p-value	Genes
Regulation of Interleukin-8 Production (GO:0032677)	5/83	4.99E-07	IL6, TNF, F3, IL6R, TLR4
Positive Regulation of Leukocyte Migration (GO:0002687)	4/28	4.99E-07	IL6, TNF, IL6R, ICAM1
Regulation of Chemokine Production (GO:0032642)	4/43	1.99E-06	IL6, TNF, IL6R, TLR4
Positive Regulation of Chemokine Production (GO:0032722)	4/54	3.81E-06	IL6, TNF, IL6R, TLR4
Positive Regulation of Interleukin-8 Production (GO:0032757)	4/63	5.73E-06	IL6, TNF, F3, TLR4
Positive Regulation of Interleukin-6 Production (GO:0032755)	4/77	1.08E-05	IL6, TNF, IL6R, TLR4
Positive Regulation of Cytokine Production Involved in Inflammatory Response (GO:1900017)	3/18	1.14E-05	IL6, TNF, TLR4
Monocyte Chemotaxis (GO:0002548)	3/23	1.79E-05	IL6, IL6R, PF4
Cytokine-Mediated Signaling Pathway (GO:0019221)	5/255	1.79E-05	IL6, TNF, F3, IL6R, PF4
Inflammatory Response (GO:0006954)	5/261	1.79E-05	IL6, VCAM1, TNF, TLR4, PF4

Table S4. Human Phenotype Ontology enrichment analysis of docking-derived thromboinflammatory targets. Enriched clinical phenotypes were identified using the Enrichr platform. Terms are ranked

according to adjusted p-values. Overlap indicates the number of genes associated with each phenotype.

Term	Overlap	Adjusted p-value	Genes
Joint hemorrhage (HP:0005261)	3/11	2.36E-06	VWF, F10, F2
Menorrhagia (HP:0000132)	3/17	4.86E-06	F10, VWF, F2
Pulmonary embolism (HP:0002204)	3/44	6.11E-05	SERPINC1, F2, TLR4
Epistaxis (HP:0000421)	3/48	6.11E-05	F10, VWF, F2
Deep venous thrombosis (HP:0002625)	2/9	1.88E-04	SERPINC1, F2
Prolonged partial thromboplastin time (HP:0003645)	2/11	2.39E-04	F10, F2
Gingival bleeding (HP:0000225)	2/20	6.81E-04	F10, F2
Prolonged bleeding time (HP:0003010)	2/21	6.81E-04	VWF, F2
Variable expressivity (HP:0003828)	3/144	7.38E-04	F10, VWF, F2
Abnormality of the pericardium (HP:0001697)	2/36	1.59E-03	IL6, TLR4

S10. Supplementary Figures

Supplementary Figures include additional fluoroscopic imaging examples supporting the representative venographic and ultrasonographic findings shown in main Figure 1, together with graphical visualizations of propensity score diagnostics, functional enrichment analyses, and phenotype association analyses.

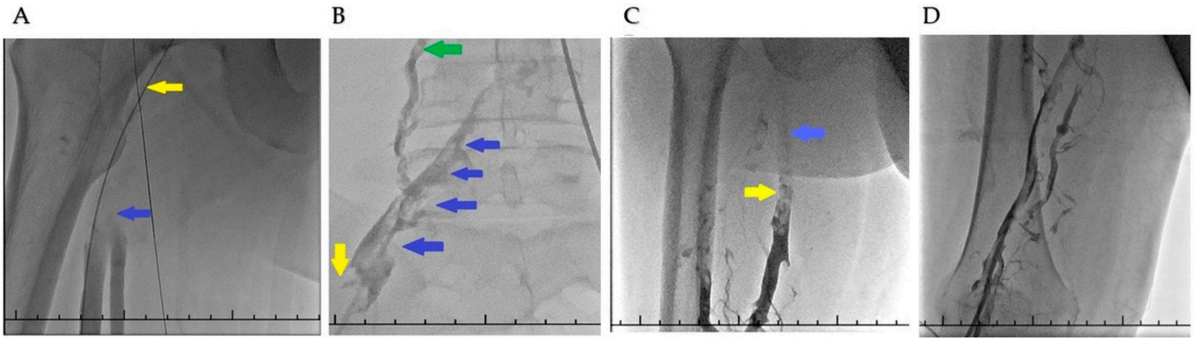

Figure S1. Additional fluoroscopic examples of venous obstruction, collateral filling, and thrombus burden in medically less responsive lower-extremity deep vein thrombosis. (A) Fluoroscopic image showing occlusive venous obstruction at the confluence of the left femoral vein and profunda femoris vein. The blue arrow indicates the obstructed venous segment with interruption of contrast progression, whereas the yellow arrow identifies the hydrophilic guidewire advanced through the

femoral venous axis toward the iliac vein during catheter-directed intervention after inadequate response to medical therapy. (B) Fluoroscopic image obtained in a patient with extensive iliofemoral deep vein thrombosis. Blue arrows highlight multiple intraluminal filling defects within the iliac venous segment, producing an irregular “moth-eaten” contrast pattern consistent with heavy thrombus burden. The yellow arrow marks the distal site of luminal compromise, whereas the green arrow indicates contrast opacification within collateral venous channels containing suspected mobile thrombotic material. (C) Fluoroscopic image demonstrating acute thrombotic obstruction within the femoral venous system in severe lower-extremity deep vein thrombosis. The blue arrow marks the level of proximal flow arrest or near-complete luminal occlusion, while the yellow arrow highlights the contrast-opacified thrombotic venous segment. Sparse collateral visualization is consistent with impaired venous outflow and insufficient symptomatic regression under LMWH therapy. (D) Additional fluoroscopic projection demonstrating extensive venous remodeling and collateral channel formation in the setting of occlusive lower-extremity venous thromboembolism. The image shows irregular contrast passage through collateralized venous pathways, supporting the presence of advanced thrombus burden and altered venous drainage.

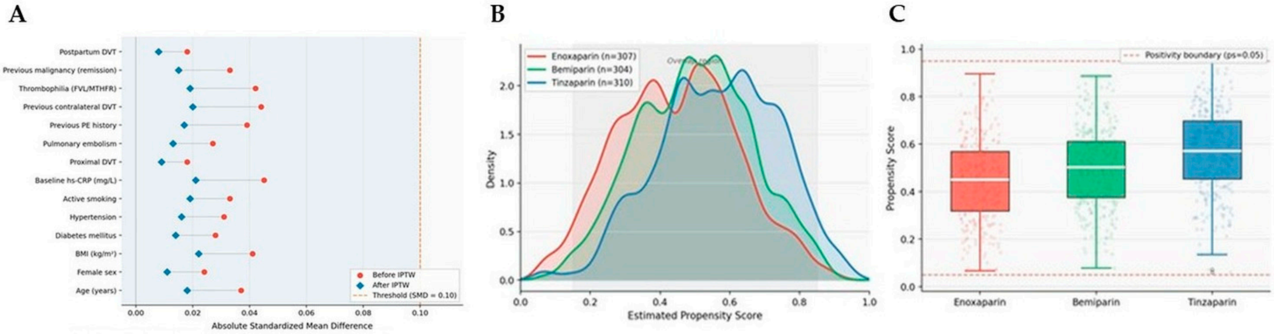

Figure S2. Propensity score diagnostic assessment following inverse probability of treatment weighting (IPTW). (A) Love plot illustrating absolute standardized mean differences (SMD) for all prespecified baseline covariates before and after weighting. The dashed vertical line indicates the prespecified SMD threshold of 0.10 for acceptable covariate balance. (B) Kernel density distributions of estimated propensity scores across enoxaparin, bemiparin, and tinzaparin treatment groups, demonstrating substantial overlap after weighting. (C) Box plots with jittered individual propensity score distributions for each treatment group, illustrating the absence of major positivity violations or extreme propensity score clustering. Dashed horizontal lines indicate prespecified positivity boundaries (0.05–0.95). Abbreviations: IPTW, inverse probability of treatment weighting; SMD, standardized mean difference; DVT, deep vein thrombosis; PE, pulmonary embolism; FVL, Factor V Leiden; MTHFR, methylenetetrahydrofolate reductase; hs-CRP, high-sensitivity C-reactive protein.

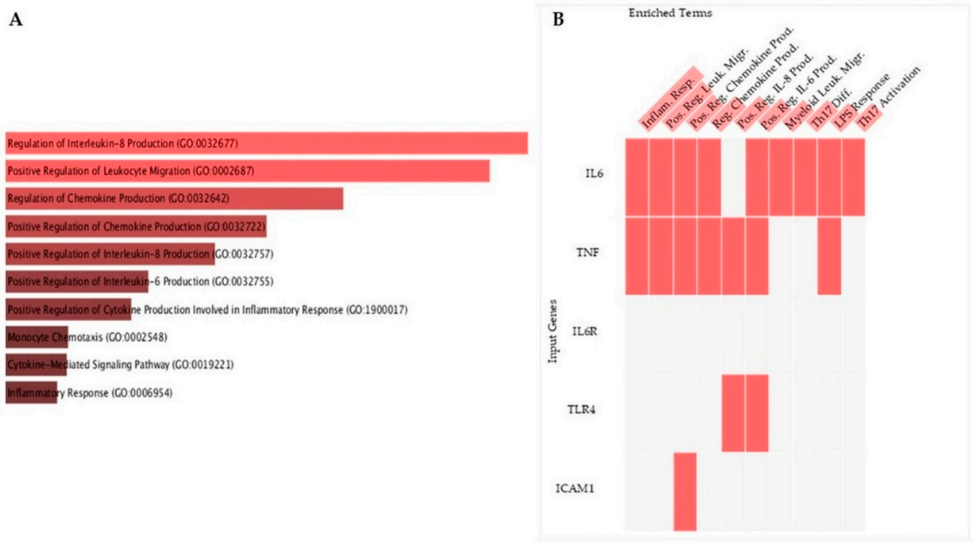

Figure S3. GO Biological Process enrichment analysis of docking-derived thromboinflammatory targets. (A) Bar plot showing significantly enriched inflammatory and immune regulatory biological processes, including regulation of interleukin-8 production, leukocyte migration, and chemokine-mediated signaling, ranked according to adjusted p-values. (B) Clustergram illustrating gene–biological process associations among cytokine signaling and inflammatory pathways. *Abbreviations*: Inflamm. Resp., inflammatory response; Pos. Reg. Leuk. Migr., positive regulation of leukocyte migration; Pos. Reg. Chemokine Prod., positive regulation of chemokine production; Reg. Chemokine Prod., regulation of chemokine production; Pos. Reg. IL-8 Prod., positive regulation of interleukin-8 production; Pos. Reg. IL-6 Prod., positive regulation of interleukin-6 production; Myeloid Leuk. Migr., myeloid leukocyte migration; Th17 Diff., T-helper 17 cell differentiation; LPS Response, response to lipopolysaccharide; Th17 Activation, T-helper 17 cell activation.

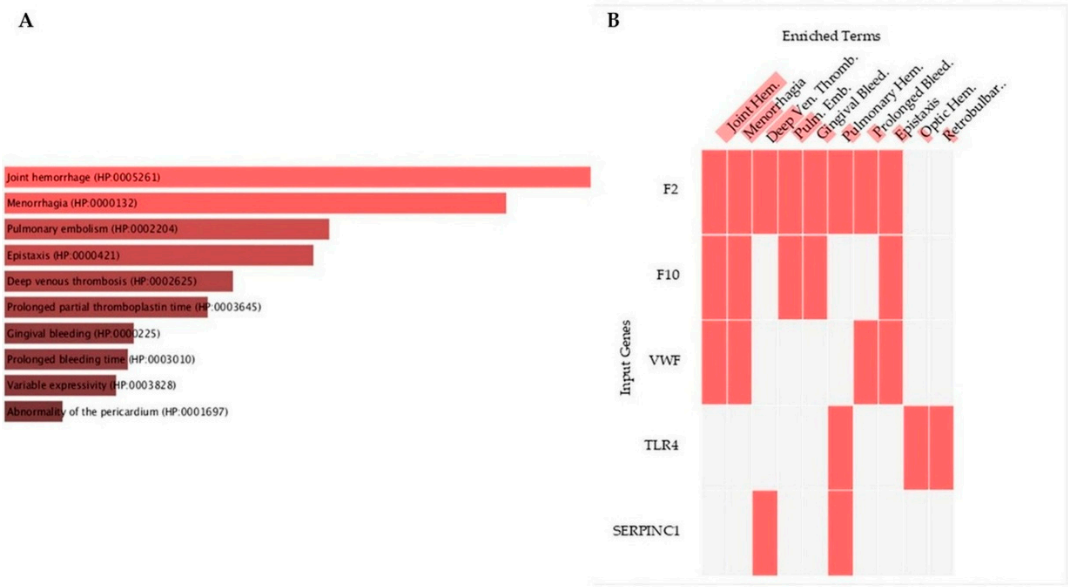

Figure S4. Human Phenotype Ontology enrichment analysis of docking-derived thromboinflammatory targets. (A) Bar plot showing clinically relevant thromboembolic and bleeding-related phenotypes, including pulmonary embolism, deep venous thrombosis, and hemorrhagic manifestations. (B) Clustergram illustrating gene–phenotype associations across enriched thromboinflammatory clinical features. *Abbreviations:* Joint Hem., joint hemorrhage; Menorrhagia, heavy menstrual bleeding; Deep Ven. Thromb., deep venous thrombosis; Pulm. Emb., prolonged pulmonary embolism; Gingival Bleed., gingival bleeding; Pulmonary Hem., pulmonary hemorrhage; Prolonged Bleed., prolonged bleeding; Epistaxis, nasal bleeding; Optic Hem., optic nerve hemorrhage; Retrobulbar Hem., retrobulbar hemorrhage.
